# Preterm birth and social support services for prenatal depression and social determinants

**DOI:** 10.1371/journal.pone.0255810

**Published:** 2021-08-13

**Authors:** Rebecca Reno, Johanna Burch, Jodi Stookey, Rebecca Jackson, Layla Joudeh, Sylvia Guendelman

**Affiliations:** 1 Center of Excellence in Maternal, Child, and Adolescent Health, School of Public Health, University of California Berkeley, Berkeley, California, United States of America; 2 Dept. of Obstetrics, Gynecology, and Reproductive Sciences, University of California San Francisco, San Francisco, California, United States of America; 3 San Francisco Department of Public Health, San Francisco, California, United States of America; Flinders University, AUSTRALIA

## Abstract

Preterm birth (PTB; <37 weeks gestation), is a leading cause of infant mortality and morbidity. Among those born preterm, risk increases as gestational age at birth decreases. Psychosocial factors such as depression symptoms and social determinants of health (SDH) may increase risk for PTB. Research is needed to understand these risk factors and identify effective interventions. This retrospective cohort study recruited English- and Spanish-speaking women presenting symptoms of preterm labor or admitted for PTB from an urban county hospital in the San Francisco Bay Area (n = 47). We used an iterative analytic approach by which qualitative data informed an exploratory quantitative analysis. Key exposures were presence of self-reported depression symptoms during pregnancy, SDH along eight domains, and receipt of behavioral health services. The outcome was gestational age at birth. T-tests, Wilcoxon rank sum tests, and linear regression models were used to test associations between the exposures and gestational age. Most participants were Black (25.5%) or Latina (59.6%). After adjusting for covariates, participants with depression symptoms had an average gestational age 3.1 weeks shorter (95% CI: -5.02, -1.20) than women reporting no symptoms. After adjusting for covariates, high number of adverse social determinants (≥ 4) suggested an association with shorter gestational age (*p* = 0.07, 1.65 weeks, 95% CI: -3.44, 0.14). Receipt of behavioral health services was associated with a significantly later gestational age; the median difference was 5.5 weeks longer for depression symptoms, 3.5 weeks longer for high social determinants, and 6 weeks longer for depression symptoms and high social determinants. Among a cohort of high-risk pregnant women, both depression symptoms during pregnancy and co-occurring with exposure to high adverse SDH are associated with shorter gestational age at birth, after controlling for psychosocial factors. Receipt of behavioral health services may be an effective intervention to address disparities in PTB.

## Introduction

Preterm birth (PTB)–a birth occurring at <37 weeks of completed gestation—is a leading cause of infant mortality and morbidity presenting emotional and economic costs to families [1]. Among preterm infants, neonatal morbidities and mortality decline as gestational age at birth increases thus making gestational age at delivery one of the major determinants of neonatal survival and morbidity [2]. In the United States, the rate of PTB has steadily increased since 2014, from 9.6% to 10.0% in 2018 [3]. Disparities in rates by race and socioeconomic status are stark. African Americans have PTB rates 50% higher than non-Hispanic white women, at 14.1% versus 9.1%; Hispanic women have slightly higher rates at 9.7% [3]. Low-income women are also at increased risk of PTB [4]; this may be associated with individual and household poverty and community conditions (i.e. neighborhoods with concentrated poverty and cumulative disadvantage) [5, 6].

Beyond socioeconomic and racial inequities, risk factors for PTB include multiple, multifaceted biomedical and psychosocial components [7]. Biomedical risks of PTB include hypertensive disease, diabetes, underweight and other medical co-morbidities, multiple gestation, inflammation and infection, uterine or cervical abnormalities, and genetic and epigenetic variants [8]. Psychosocial risks involve mental health conditions such as depression and anxiety, perceived stress, traumatic events, lack of social supports, and unmet social needs or social determinants of health (e.g. low levels of education, housing, transportation and food insecurity) [9–11]. Social determinants of health are those conditions in the places where people are born, grow, live, learn, work, and play that may make them vulnerable to poor health [12].

Current social-ecological frameworks of social determinants of health posit that individual lifestyles and behaviors are embedded in living and working conditions and in social norms and networks, which in turn, are related to the wider socio-economic and cultural environment. The combined effect of multi-layered, inter-related risk factors that often co-occur, generate social inequities in health in the U. S. [13, 14]. Evidence suggests increased exposure to adverse social determinants, more likely seen among African American and U. S. born Latina women, perpetuate disparities in birth outcomes [15]. For these women, risk factors are heightened by structural and interpersonal racism, immigration stress, and disenfranchisement [16–19]. Biological models posit that exposures to chronic and repeated stress across the life course from psychosocial factors and structural inequities increase allostatic load, which may influence birth outcomes through physiological pathways such as placental function [20–22].

Women experiencing complex risk profiles stemming from adverse social determinants and mental health conditions may benefit from behavioral health services (i.e. seeing a social worker or counselor) during pregnancy [23]. A recent Cochrane systematic review of randomized trials of social support during pregnancy shows that compared to routine care, programs offering additional emotional, informational, or instrumental/tangible support reduce the risk of PTB slightly, although not significantly (RR = 0.92; 95% CI: 0.84, -1.01) [24]. However, this null effect may be due to the differing criteria for participants’ “high risk for PTB” status across studies. A 2019 U.S. Preventive Task Force recommends clinicians provide or refer pregnant and postpartum women who are at increased risk of perinatal depression to counseling intervention [25]. In the absence of a validated screening tool for identifying at-risk women, the task force notes that women should be considered at-risk if they have a history of depression, current depressive symptoms, anxiety, or exposure to adverse social determinants such as low income or single parenthood, or are experiencing intimate partner violence and negative life events [26].

Studies that examine the association between depressive symptoms and gestational age have shown inconsistent results [27–29]. Furthermore, we could not find published studies that examined the contribution of both depression symptoms and social determinants on preterm birth. Prior studies often capture social determinants as demographic characteristics (e.g. income and education) included as control variables when measuring the association between mental health and preterm birth, or when measuring the association between neighborhood-level factors and preterm birth [30, 31]. We postulated that depression symptoms and exposure to multiple adverse social determinants were associated with gestational age at birth. We further hypothesized that receipt of behavioral health services could protect against early gestational age at birth. This is an important policy issue, given the need to identify entry points for reducing or eliminating the synergistic effects of psycho-social risk factors on poor health outcomes. To test these hypotheses, we conducted an exploratory mixed-methods study of psychosocial risks and behavioral health services which aimed to: 1) examine the unique and co-occurring associations between social determinants of health and depression symptoms and gestational age at birth and 2) assess the potential benefit of receipt of behavioral health services on gestational age.

## Methods

The San Francisco Preterm Birth Review (PTBR) is a retrospective cohort study and public health initiative aimed at developing a feasible public health surveillance system for preterm birth in San Francisco, CA. Data collection tools were designed using the Dahlgren and Whitehead rainbow model, which is a social-ecological framework that positions health as a function of individual constitutional factors, lifestyles, and behaviors embedded within upstream social, community, cultural, and environmental factors [13]. PTBR enrolled patients from an urban safety net hospital ranked as having the second highest Black singleton preterm birth rate in the city (12.6%, compared with a county-wide rate of 6.3% (95% CI = 1.0–6.6)) [32]. Patients were enrolled if they were either in labor and delivery triage or during an antepartum admission for spontaneous preterm labor, had premature rupture of membranes (PROM) or hypertensive disorder, or had a preterm birth. Study personnel collected in-depth medical, obstetric, mental health, and social risk and protective factors for PTB from participants.

For this study, quantitative and qualitative methods were combined using an iterative analytic approach [33]. At the outset of our study we sought to ground our research in participants’ lived experiences, and designed our analyses to be responsive to those issues highlighted by the participants themselves. To this end, we reviewed the qualitative data collected through semi-structured interviews conducted in English and Spanish. These interviews had been coded in Dedoose (v.8.0.35) using grounded theory by two study team members. Interrater reliability using pooled Cohen’s kappa coefficient was 1.0, which surpassed the pre-determined threshold of 0.7.

In a review of the qualitative data the study team noted clusters of social determinants that often co-existed with symptoms of depression, and were sometimes mitigated by the receipt of behavioral health services. This guided the development of our quantitative study hypotheses and selection of study variables. To inform our quantitative analysis we reviewed each quote linked to relevant themes/codes including “social determinants of health,” “mental health symptoms,” and “access to social support services,” and examined the correspondence between these codes and survey questions.

### Participants

English or Spanish-speaking pregnant women, 18 years and older, with viable pregnancies 24 weeks gestation or beyond, were recruited from the urban safety net hospital between October 2017 and March 2019. Trained research assistants or public health nurses conducted semi-structured interviews, administered written or spoken surveys, and/or completed a chart abstraction of electronic medical records (EMR) and birth certificates. Participants provided separate written consent for each component of the study as well as for collection of information about mental health, substance use, and HIV. Some participants gave consent to collect prenatal care information but not labor and delivery and vice versa. A $50 incentive was provided for participation in the study. All study procedures were approved by the University of California, San Francisco Institutional Review Board (Study ID: 17–21932).

Out of the 121 women recruited for the study, 73 consented to participate. Nineteen study participants were lost to follow-up prior to delivery at the study hospital, or did not consent to EMR abstraction or birth certificate review and thus did not have gestational age data. From the remaining 54 participants, we excluded participants delivering twins (n = 2), and participants with missing information on depression symptoms or treatment (n = 5). The final analytic sample comprised 47 participants. A comparison of participants with missing data on depression symptoms, treatment, or gestational age versus participants with complete data showed no significant differences by age, race, education, family income, parity, or number of adverse social determinants.

The survey was designed using a social ecological framework with questions focused on individual, organizational, and community-level determinants. Participants were queried about individual risk factors and psychosocial stressors including mental health history, depression symptoms, and experiences related to social determinants of health. The survey was completed either in person or by phone, in English or Spanish. Survey administration took place after delivery at the hospital before discharge of the baby from the nursery, at an outpatient clinic visit, or at the participant’s home up to three weeks after hospital discharge.

### Measures

The main outcome of interest was gestational age at birth (in completed weeks) obtained from the EMR based on best obstetric estimate early in pregnancy. This measure was examined both as a continuous and a categorical variable (extremely preterm [<28 completed weeks], moderate preterm [28–33 weeks], late preterm [34–36 weeks], and term [≥ 37 completed weeks]), using the World Health Organization’s preterm categories. Term births occurred among women who were enrolled during an antepartum admission for preterm labor, PROM, or hypertension and went on to deliver at term.

Key exposures were the presence (or absence) of depression symptoms during pregnancy and the median number of adverse social determinant of health domains. We measured these exposures using the study questionnaire. This questionnaire was not validated prior to administration, however whenever possible validated questions were selected from other surveys, with a priority on selecting measures that allowed a comparison of the study sample to city-, county- or state-wide data. Presence (or absence) of depression symptoms was derived from a survey question adapted from the Patient Health Questionnaire-2 [34]–“During your pregnancy, did you ever have 2 weeks or longer when you felt sad, empty, or depressed for most of the day?” Responses were treated as dichotomous.

**Fig 1:**
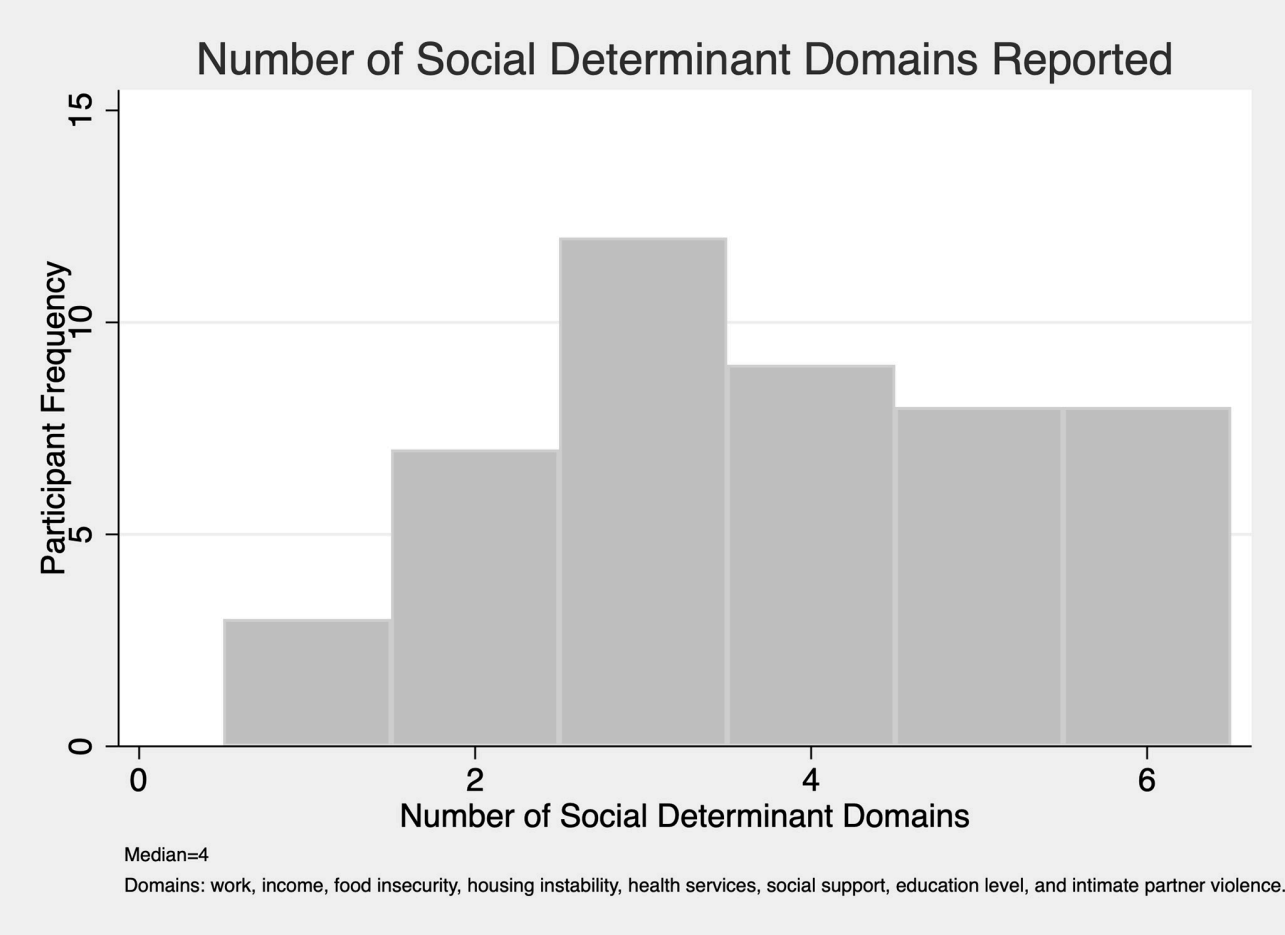
Social determinant domain frequencies (n = 47).

Social determinants consisted of eight domains constructed from survey items that measured: employment, economic issues, food security, housing stability, access and acceptability of health care, social support, educational level, and intimate partner violence. The number of adverse items in each domain ranged from one to seventeen (S1 Table). We dichotomized each domain; participants who had one or more of the items within a given domain were coded as having exposure to that adverse social determinant. As there is a lack of research literature establishing a validated cut point for exposure to a number of adverse social determinants, we established the median number of social determinant domains experienced by participants and created two groups: <4 and ≥ 4 adverse social determinants (Fig 1). We identified clusters of social determinants through the creation and examination of a table listing every co-occurring social determinant combination and their associated frequency.

We measured receipt of behavioral health services with two survey questions. The first asked respondents to indicate whether they had seen a counselor for personal or family problems, stress, depression, or anxiety during pregnancy. The second queried respondents whether they had received help from a social worker in a clinic during pregnancy. We created a single dichotomous variable to capture whether participants saw either a social worker or a counselor during pregnancy.

Additional covariates encompassed demographic, health, and behavioral health characteristics. Demographic characteristics abstracted from the EMR and/or birth certificate included race, age, income, education, and parity. Health characteristics abstracted from the EMR included medical co-morbidities (gestational diabetes, hypertensive disorders, and obesity), prior preterm birth, and receipt of medical interventions (cerclage and vaginal progesterone) for preventing PTB. Behavioral health characteristics included depression or anxiety prior to pregnancy, and exposure to adverse childhood experiences defined as having experienced verbal, physical, emotional, and/or sexual abuse up to age 18, and whether it was hard for their family to pay for basic needs (i.e. food and housing).

### Analysis

We performed bivariate analyses to characterize the study population by the two exposures—presence of depression symptoms during pregnancy and adverse social determinants of health—using Fisher’s exact tests. Associations between presence of depression symptoms or adverse social determinants during pregnancy and continuous gestational age at birth were assessed using t-tests; associations with preterm birth as a categorical variable were evaluated with Fisher’s exact tests given the small sample size and expected cell count. To measure the association between receipt of behavioral health services and gestational age among exposed and unexposed groups, we used a Wilcoxon rank-sum test to examine the difference between medians, as it is less sensitive to outliers in gestational age. The decision to retain outliers was made *a priori* given the study aims and the anticipated variability in number of weeks gestation.

We used linear regression to identify the main correlates of gestational age. We constructed separate adjusted models to assess the relationship between i) depression symptoms, ii) social determinants of health, and iii) co-occurring depressions symptoms and adverse social determinants and gestational age. All three models controlled for receipt of behavioral health services and other covariates. Given the small sample size (n = 47) and exploratory nature of our study, covariates were initially selected for each model if they were associated (*p* value ≤ 0.10) with the outcome and/or the key independent variables in the bivariate analysis. We used a backwards elimination methodology, initially including all variables with *p* value ≤ 0.10 with the exception of federal poverty level, as income is a social determinant domain and inclusion in models examining the social determinant-gestational age relationship would result in over-adjustment. We then eliminated variables one at a time, beginning with those with the highest *p* values. If the removal of any variable resulted in a change in beta coefficients greater than 10%, those variables were retained. We present results from the regression models as coefficient estimates and their 95% confidence intervals (CI). We also conducted sensitivity analyses with race, with known medical contributors associated with preterm birth, and with interventions to extend gestational age in the models. We were underpowered to perform interaction tests to assess whether receiving behavioral services changed the relationship between exposure to depression and social determinants and gestational age. However, we stratified the two exposures by receipt of behavioral health services to examine its potential effects. The small sample size also precluded us from stratifying by race. We performed all quantitative analyses using STATA SE version 15.0 (College Station TX, Stata Corp). We supplemented quantitative data with qualitative quotes to contextualize the study findings.

## Results

### Characteristics of study population by prenatal depression symptoms and adverse social determinants

All women received services at the same hospital and in the month prior to pregnancy were either uninsured (17.0%) or received some form of public health insurance (83.0%). While 55.0% of the participants were below the federal poverty level, the highest per capita income was $40,250. The majority of participants were Black (25.5%) or Latina (59.6%), and between 25–34 years of age (46.8%). Approximately 70% were multiparous, with 14.9% reporting a prior preterm birth. More than 1 in 4 participants reported depression symptoms during pregnancy.

*So I had some sort of depression. And, for some time, I couldn’t go… to the appointments. I was very sleepy, I wouldn’t go. Sometimes I would and sometimes I wouldn’t. I wasn’t taking care of myself like I should*.

[Participant 9, Spanish-speaking]

Compared with participants who had no symptoms, those with depression symptoms were more likely to have some (incomplete) college education (*p* = 0.10), a prior history of depression or anxiety (*p* = 0.02) and experienced abuse during childhood (*p* = 0.10) (Table 1).

**Table 1:** Distribution of demographic, health and behavioral health characteristics by depression symptoms and social determinants (n = 47).

	Depression Symptoms During Pregnancy					Social Determinants During Pregnancy				
	No Depression Symptoms		Depression Symptoms		Fisher’s *p-*value	<4 Adverse Social Determinants††		≥4 Adverse Social Determinants††		Fisher’s
	n = 34 (72.3%)		n = 13 (27.7%)							
						n = 22 (46.8%)		n = 25 (53.2%)		*p-*value
Demographic Variables	n	%	n	%		n	%	n	%	
Race										
Black	6	17.7	6	46.2	0.13	3	13.6	9	36.0	0.23
Latina	22	64.7	6	46.2		15	68.2	13	52.0	
Other	6	17.7	1	7.7		4	18.2	3	12.0	
Age (years)										
< 25	5	14.7	3	23.1	0.82	1	4.6	7	28.0	0.12
25–34	17	50.0	5	38.5		12	54.6	10	40.0	
>35	11	32.4	5	38.5		8	36.4	8	32.0	
Missing	1	2.9	0	0.0		1	4.6	0	0.0	
Income										
Below FPL	13	38.2	9	69.2	0.24	5	22.7	17	68.0	0.01
Above FPL	15	44.1	3	23.1		12	54.6	6	24.0	
Missing	6	17.7	1	7.7		5	22.7	2	8.0	
Education										
0-11th grade	10	29.4	2	15.4	0.10	4	18.2	8	32.0	0.71
12th grade, no diploma	0	0.0	0	0.0		0	0.0	0	0.0	
High school graduate or GED	4	11.8	1	7.7		3	13.6	2	8.0	
Some college credit, no degree	5	14.7	7	53.9		6	27.3	6	24.0	
Associates degree	1	2.9	1	7.7		0	0.0	2	8.0	
Bachelor’s or higher degree	5	14.7	0	0.0		3	13.6	2	8.0	
Missing	9	26.5	2	15.4		6	27.3	5	20.0	
Parity										
Multiparous	24	70.6	9	69.2	1.00	15	68.2	18	72.0	0.20
Primiparous	8	23.5	3	23.1		4	18.2	7	28.0	
Missing	2	5.9	1	7.7		3	13.6	0	0.0	
Health Characteristics & Medical Interventions										
Prior preterm birth										
Yes	4	11.8	3	23.1	0.56	4	18.2	3	12.0	0.54
No	29	85.3	10	76.9		17	77.3	22	88.0	
Missing	1	2.9	0	0.0		1	4.6	0	0.0	
Gestational diabetes										
Yes	7	20.6	1	7.7	0.55	6	27.3	2	8.0	0.05
No	25	73.5	12	92.3		14	63.6	23	92.0	
Missing	2	5.9	0	0.0		2	9.1	0	0.0	
Hypertensive disorders										
Yes	10	29.4	7	53.9	0.24	9	40.9	8	32.0	0.24
No	22	64.7	6	46.2		11	50.0	17	68.0	
Missing	2	5.9	0	0.0		2	9.1	0	0.0	
Obesity										
Yes	5	14.7	3	23.1	0.83	4	18.2	4	16.0	0.40
No	27	79.4	10	76.9		16	72.7	21	84.0	
Missing	2	5.9	0	0.0		2	9.1	0	0.0	
Substance Use										
Yes	5	14.7	3	23.1	0.83	2	9.1	6	24.0	0.31
No	16	47.1	6	46.2		10	45.5	12	48.0	
Missing	13	38.2	4	30.8		10	45.5	7	28.0	
Cerclage or Progesterone										
Yes	1	2.9	2	15.4	0.18	1	4.6	2	8.0	1.00
No	33	97.1	11	84.6		21	95.5	23	92.0	
Missing	0	0.0	0	0.0		0	0.0	0	0.0	
Behavioral Health Characteristics										
History of depression or anxiety										
Yes	4	11.8	6	46.2	0.02	1	4.6	9	36.0	0.01
No	30	88.2	7	53.9		21	95.5	16	64.0	
Missing	0	0.0	0	0.0		0	0.0	0	0.0	
Adverse childhood experiences (ACE)										
Basic needs met during childhood										
Yes	20	58.8	6	46.2	0.52	15	68.2	11	44.0	0.09
No	14	41.2	7	53.9		7	31.8	14	56.0	
Missing	0	0.0	0	0.0		0	0.0	0	0.0	
Abused during childhood (verbal, physical, sexual, emotional)										
Yes	9	26.5	7	53.9	0.10	17	77.3	14	56.0	0.11
No	25	73.5	6	46.2		5	22.7	11	44.0	
Missing	0	0.0	0	0.0		0	0.0	0	0.0	

*Note: Fisher’s exact test was used given small sample size and expected cell count.

^†^ Answered ’no’ to having depression symptoms for two weeks or longer.

^††^ Represents social determinant domains which include: employment, economic issues, food security, housing stability, access and acceptability of health care, social support, educational level, and intimate partner violence.

FPL = Federal Poverty Level; GED = General Educational Development

The possible range of participants’ exposure to adverse social determinants was 1–8; the sample range was 1–6. Twenty five of the participants (53.2%) reported ≥ 4 social determinants (Fig 1). Although the clustering of social determinants varied widely, the two most frequent clusters included: 1) issues with employment, access and acceptability of health care, and low education level, and 2) employment, economic issues, food insecurity, housing instability, access and acceptability of health care, and low social support. These social determinants spanned socio-ecological levels and included individual-, organizational-, and community-level factors. One woman described the impact of violence on her family, and in her community:

*It’s just been a lot of shooting in my neighborhood. So that’s stressful, period. Especially with having kids and running around this neighborhood…well, my stepson, he got shot 27 times, but it’s just been a whole bunch of shootings in the neighborhood. It’s like at least once a week somebody gets shot or shot at*.

Participant 7, English-speaking

What is most striking related to social determinants was the participants navigation of a complex constellation of risk factors. One participant describes her circumstances:

*Since New Year’s, our car got towed for outstanding tickets. No, first, our car alternator went out. And then our car tie-rod went out…And then we got that fixed, and then my cousin passed away… Our housing situation—we stay in the shelter, but it’s more like a hotel, because we get our own room and bathroom. We got a subsidy and so I just been looking for housing; but it’s been really hard to find someone for us to rent to. That and trying to—job-wise—so we could have more income. Because our income, monthly, doesn’t really last us through the month. So, that, too*.

Participant 10, English-speaking

Compared with participants with fewer than 4 adverse social determinants, those with more adverse social determinants were more likely to fall below the federal poverty level (*p* = 0.01) and had a history of depression or anxiety (*p* = 0.01). They were less likely to have gestational diabetes (*p* = 0.05) and to report that their families faced financial hardships in covering basic needs during childhood (*p* = 0.09) (Table 1).

### Association between prenatal depression symptoms and gestational age

The gestational age at delivery of study participants ranged from 24 to 40 weeks. The distribution was negatively skewed; the majority of births (55.3%) had a gestational age of 35 weeks or later, and four outliers (11.8%) were identified with gestational ages as early as 24 weeks. As Table 2 shows, participants with depression symptoms during pregnancy had a mean gestational age of 32.9 weeks (SD = 1.02) which was 2.0 weeks shorter than those with no depression symptoms (mean 34.9 weeks, SD = 2.8).

**Table 2:** Gestational age by depression symptoms and social determinants (n = 47).

	Continuous Gestational Age		Categorical Gestational Age								
	Gestational age-weeks (mean ± SD)	t-test *p-*value	Extremely preterm (<28 weeks)		Moderate preterm (28–33 weeks)		Late preterm (34–36 weeks)		Term (≥37 weeks)		Fisher’s *p-*value
			n	%	n	%	n	%	n	%	
Depression symptoms only											
Depression symptoms during pregnancy n = 13 (27.7%)	32.92 ± 1.02	0.05	1	7.7	4	30.8	6	46.2	2	15.4	0.44
No depression symptoms during pregnancy† n = 34 (72.3%)	34.94 ± 2.83		1	2.9	5	14.7	20	58.8	8	23.5	
Adverse social determinants of health (SDH) only††											
≥4 adverse SDH n = 25 (53.2%)	33.76 ± 3.91	0.15	2	8.0	6	24.0	10	40.0	7	28.0	0.12
<4 adverse SDH n = 22 (46.8%)	35.09 ± 1.93		0	0.0	3	13.6	16	72.7	3	13.6	
Depression symptoms and adverse social determinants											
Co-occurring depression symptoms, ≥ 4 adverse SDH n = 12 (25.5%)	32.92 ± 3.85	0.06	1	8.3	3	25.0	6	50	2	16.7	0.73
No co-occurring depression symptoms, ≥ 4 adverse SDH n = 35 (74.5%)	34.89 ± 2.81		1	2.9	6	17.1	20	57.1	8	22.9	

*Note: Fisher’s exact test was used given small sample size and expected cell counts.

^†^ Answered ’no’ to having depression symptoms for two weeks or longer.

^††^ Represents social determinant domains which include: employment, economic issues, food security, housing stability, access and acceptability of health care, social support, educational level, and intimate partner violence.

These findings were confirmed in the unadjusted linear regression model (b = -2.02; 95% CI: -4.04, -0.01) and became even stronger when controlling for abuse during childhood and seeing a social worker or counselor (b = -3.11; 95% CI: -5.02, -1.20) (Table 3). Sensitivity analyses showed that when we included race, medical risk factors (i.e. pre-pregnancy hypertension or preeclampsia, gestational diabetes, and prior preterm birth), and medical interventions to extend gestational age (i.e. cerclage and progesterone) in separate models, the results did not significantly differ from the final adjusted model reported.

**Table 3:** Regression models examining associations between gestational age and prenatal depression symptoms, adverse social determinants, and co-occurring depression symptoms and social determinants.

	Unadjusted Model*			Adjusted Model*		
	coefficient estimate	95% CI	*p*	coefficient estimate	95% CI	*p*
Model 1: Depression symptoms only						
No depression symptoms	Reference			Reference		
Depression symptoms	-2.02	-4.04, 0.01	0.05	-3.11	-5.02, -1.20	<0.01
Saw a social worker or counselor†	-	-	-	2.24	0.57, 3.91	0.01
Physical, emotional, or sexual abuse during childhood†	-	-	-	2.08	0.31, 3.85	0.02
Model 2: Adverse social determinants of health (SDH) only††						
<4 adverse SDH	Reference			Reference		
≥4 adverse SDH	-1.33	-3.18, 0.52	0.15	-1.65	-3.44, 0.14	0.07
Saw a social worker or counselor†	-	-	-	2.06	0.27, 3.86	0.03
Model 3: Depression symptoms and adverse SDH						
Co-occurring depression symptoms, ≥ 4 adverse SDH	Reference			Reference		
No co-occurring depression symptoms, ≥ 4 adverse SDH	-1.97	-4.05, 0.11	0.06	-3.06	-5.05, -1.07	<0.01
Saw a social worker or counselor†	-	-	-	2.08	0.41, 3.75	0.02
Physical, emotional, or sexual abuse during childhood†	-	-	-	2.18	0.37, 3.99	0.02

*The sample size for all unadjusted models was n = 47. The sample size for the adjusted models for depression symptoms, adverse social determinants, and co-occurring depression symptoms and adverse social determinants was n = 47, n = 40, and n = 40 respectively. Sample sizes differ due to missing data on adjustment variables. Adjustment variables were selected separately for each model on the basis of their association with the exposure and/or outcome, and if their removal in the model resulted in a change in beta coefficients greater than 10%.

^†^ Indicates an adjustment variable.

^††^ Represents social determinant domains which include: employment, economic issues, food security, housing stability, access and acceptability of health care, social support, educational level, and intimate partner violence.

#### Association between social determinants and gestational age

On average, participants with 4 or more adverse social determinants had a gestational age that was 1.3 weeks shorter than those with fewer adverse social determinants (Table 2), although this relationship was not significant in the unadjusted regression model (b = -1.33; 95% CI: -3.18, 0.52) (Table 3). Notably, the relationship between adverse social determinants and shorter gestational age approached significance (*p* = 0.07) after adjusting for seeing a social worker or counselor (b = -1.65; 95% CI: -3.44, 0.14). Further sensitivity analyses showed that when we included race, medical risk factors (i.e. pre-pregnancy hypertension or preeclampsia, gestational diabetes, and prior preterm birth), and medical interventions (i.e. cerclage and vaginal progesterone) in separate models, the results did not significantly differ from the final adjusted model reported.

### Co-occurring depression symptom and high exposure to social determinants and gestational age

Compared to those without co-occurring depression symptoms and high exposure to adverse social determinants, participants experiencing both depression symptoms and more than four adverse social determinants had gestational ages at birth that were shorter by 3.06 weeks (95% CI: -5.05,-1.07), after adjustment for seeing a social worker or counselor, and having experienced physical emotional, or sexual abuse during childhood (Table 3).

The adjusted regression models also indicated that exposure to abuse during childhood was positively associated with gestational age. The majority of participants who experienced abuse were hospitalized for preterm labor and saw their gestational age at birth increase. Sensitivity analysis showed that when we included race, pre-pregnancy hypertension or preeclampsia, gestational diabetes, prior preterm birth, and medical interventions (i.e. cerclage and vaginal progesterone) in separate models, the results did not significantly differ from the final adjusted model reported.

### Association between behavioral health services and gestational age

In the interviews, participants reported receiving behavioral health services from a range of provider types including social workers who provided mental health and social service support, and counselors, psychologists, or other mental health professionals. One participant explains how receipt of these services helped her manage her circumstances:

*They [prenatal clinic] offered me a social worker. So, the social worker asked me many questions like—the same questions of the survey: if I was having problems, needed food, needed emotional support. And so, she offered it to me. If I needed, she could put me in contact with services*.

[Participant 3, Spanish-speaking]

For several participants, issues related to social determinants were the vehicle through which they were able to access mental health services, though often they were added to waitlists or engaged through a complex referral process:

*It started because I had got an eviction notice at my apartment. Because I was like, late on my rent. And then I got hooked up with [nonprofit] to help me with my eviction case stuff. And then my lawyer there referred me to somebody…and so I was seeing somebody temporarily. And as I’m meeting with her, she was finding other places for me—putting me on the wait list for places like [mental health clinic]. I’m a part of them. That’s where I get my therapy*.

[Participant 39, English speaking]

**Fig 2:**
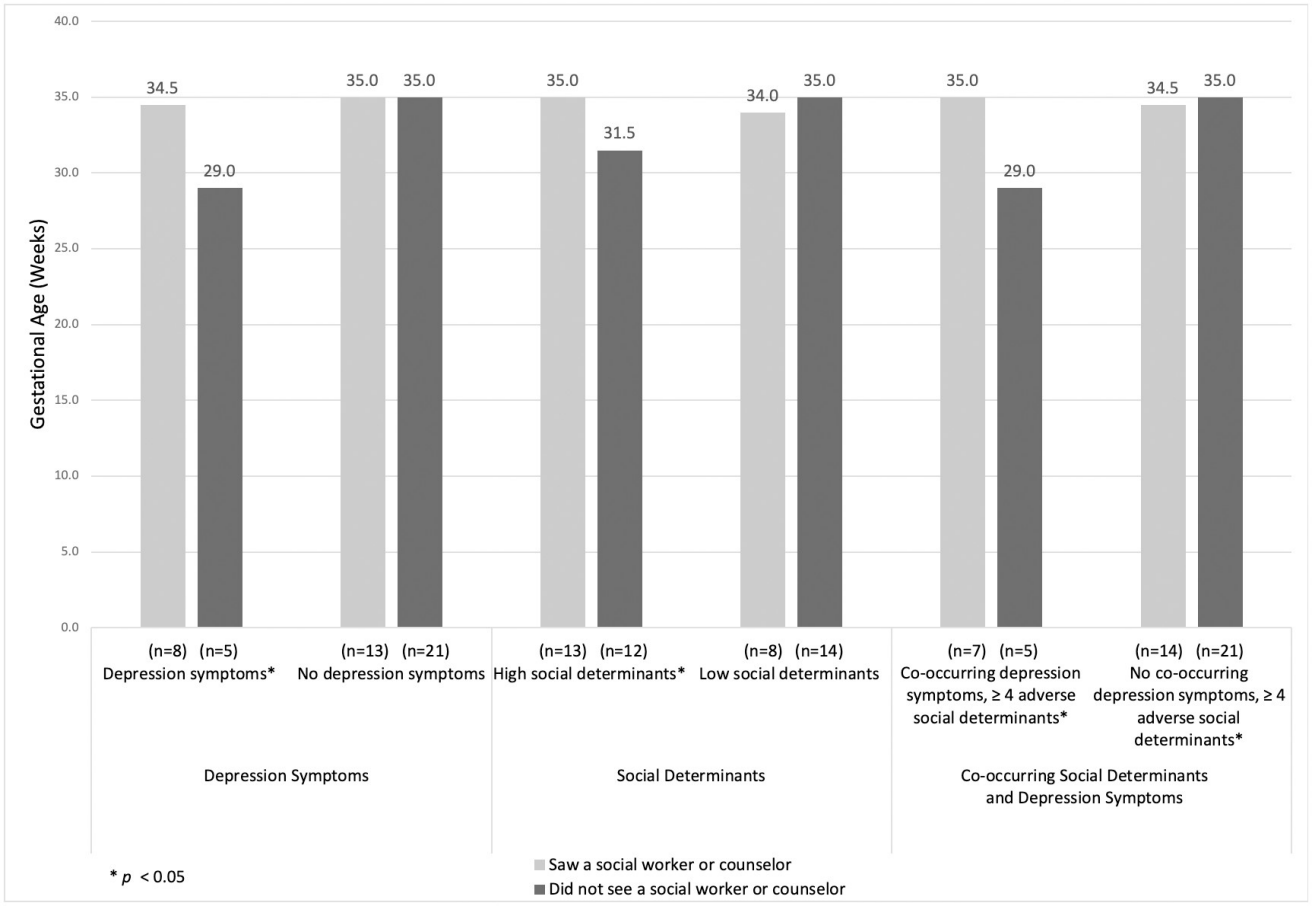
Differential associations between behavioral health services and gestational age for selected characteristics (n = 47).

Fig 2 shows the association between gestational age at birth among participants with and without depression symptoms and/or with high or low social determinants, stratified by whether or not they received services from a behavioral health provider. For participants with depression symptoms during pregnancy, seeing a social worker or counselor was associated with a much later gestational age at birth (34.5 vs. 29.0 weeks; *p* = 0.01). In contrast, for participants without depression symptoms during pregnancy, seeing a behavioral health provider was not associated with gestational age at birth. Similarly, the association between receipt of behavioral health services and gestational age was significant among those with four or more adverse social determinants (35.0 vs. 31.5; *p =* 0.02), but a relationship was not found among those with fewer than four adverse social determinants. One in four participants experienced depression symptoms coupled with exposure to a high number of adverse social determinants, constituting the most high-risk psychosocial group. For these participants, seeing a social worker or counselor was associated with a 6 week longer gestational age (difference in medians), which was statistically significant (*p* = 0.01). The association between seeing a behavioral health provider and a longer gestation at birth was also found in the adjusted models (Table 3).

## Discussion

Our exploratory study of women at high risk for, or having experienced preterm birth found evidence of associations between depression symptoms and depression symptoms co-occurring with high exposure to adverse social determinants—and shorter gestational age at birth. The evidence for the association of social determinants of health and gestational age was suggestive of a relationship in the adjusted model. These findings are important because gestational age at delivery is one of the major determinants of neonatal survival and morbidity. Manuck et al. found that among infants born between 26 and 32 weeks gestation in 2008–2011, each additional week in utero was associated with a lower incidence of major neonatal morbidity and a minimum of eight fewer days of hospitalization for the infant [2].

Published studies measuring associations between depression and gestational age are inconsistent due to small sample sizes, differing ways of assessing depression symptoms and/or diagnoses, and differences in study designs; furthermore, studies often do not assess receipt of behavioral health or social support services. A recent systematic review of depression (inclusive of studies using validated screening tools measuring self-reported symptoms, structured psychiatric interviews, or a chart review capturing current unipolar depression diagnosis) and birth outcomes found that only three of fourteen studies showed statistically significant associations between depression and gestational age [27]. Nonetheless, at least two recent studies measuring sub-clinical depression (i.e. depression symptoms not meeting clinical criteria) show an association with shorter gestational age or preterm birth. Van Dijk et al. [28] assessed 4,044 women and found that experiencing depression symptoms during pregnancy categorized as ‘major’ was associated with shorter gestational age (*p* = 0.02). Fransson, Örtenstrand, and Hjelmstedt [29] concluded that even moderate levels of prenatal depressive symptoms significantly increased the risk of preterm birth.

Our findings are consistent with much of the research on the impact of social determinants of health on birth outcomes. One systematic review of 106 studies found that 93 supported a significant association between socioeconomic disadvantage and adverse birth outcomes including preterm birth [4]. In one cross-sectional study using birth certificate data focused on Black-white disparities in preterm birth, researchers found that maternal education level, marital status/paternal acknowledgement, and source of payment for delivery were the largest contributing factors [15]. We could not find published studies that examined the co-occurring associations between depression symptoms and social determinants on PTB, which our study demonstrates.

Notably, our findings show that among participants with depression symptoms during pregnancy and/or exposure to multiple adverse social determinants, receipt of behavioral health services during pregnancy may mitigate these psychosocial risks in so far as the intervention is associated with a longer gestational age. This finding was not demonstrated for the group with low or no psychosocial risks. These findings may reconcile null and inconsistent effects in the published literature, particularly in studies where the sample is not stratified by risk. Although our small sample precluded us from conducting interaction tests to assess effect modification of behavioral health service receipt, the findings lead us to hypothesize that among participants with preterm delivery who additionally experience significant psychosocial stressors, behavioral health services likely influence the pathways between social determinants and PTB and between mental distress and PTB.

Current public health systems for pregnancy and childbirth prioritize medical care over non-medical support [35]. Our study findings suggest that pregnant women at risk of preterm delivery who experience mental health distress and multiple social determinants must be identified and offered behavioral health services (i.e. counseling or social work services) to improve their birth outcomes. These findings corroborate the recent recommendations provided by the U.S. Prevention Health Task Force [22]. While sources of social support such as doulas, community health workers, family members or group prenatal care may be effective interventions, less is known about the role and impact of social workers or counselors in prenatal and childbirth settings [36]. Additional research is needed to better understand the availability, accessibility, acceptability, utilization, and impact of behavioral health services, particularly among pregnant low income women and women of color [37, 38]. Patient-centered care that routinely incorporates social risk and mental health screenings into care decisions, as is recommended by the American College of Obstetrics and Gynecologists [39, 40], should be promoted.

Interventions are needed to help at-risk pregnant women navigate their social determinants and support their mental health; however, to make durable improvements in preterm birth inequities, we must also change the institutions and policies that place certain groups at higher risk in the first place. What underlies risk, particularly for Black and Latina women is not the fact that they are non-white, rather it is what it means to be non-white in the U.S. [41]. Social determinants of health, such as those that are measured in this study, are a consequence of the legacy of racism (both interpersonal and structural). Sustainable change requires investing in communities [5, 6], as concentrated poverty may mediate associations between mental health and preterm birth [30]. It also requires investing in social services, structures, and institutions, as state-level investments in these areas are associated with better health outcomes [42].

### Strengths and limitations

Our study has a number of notable strengths. First, the mixed methods design enabled us to gather in-depth qualitative and quantitative data from what is often a difficult to reach population—low-income Black and Hispanic women experiencing preterm labor, preterm birth, and medical/obstetric issues that can result in medically-indicated preterm birth. The PTBR survey captured the breadth of multiple adverse social determinants of health, and the grounded approach of our study incorporated participants’ lived experiences to contextualize their exposure to social determinants, mental health, and receipt of behavioral health services. The survey tool included several questions that consistently have been used in representative county- and state-wide surveys, allowing us to compare data. Although our sample prenatal depression rate at 27.7% was higher than county-wide (14.4% (95% CI 10.8–17.9)) and state-wide rates (14.1% (95% CI 13.1–15.0)), our study sample prevalence rate is comparable to county-wide statistics when disaggregated by race and insurance type [43, 44]. In San Francisco County 21.4% (95% CI 14.0–28.9) of Black and 26.7% (95% CI 16.6–36.7) of Hispanic respondents reported depression symptoms [44]. Further, in San Francisco among women with Medi-Cal insurance 24.1% reported prenatal depressive symptoms [45]. Such similarities in prevalence rates indicate that prenatal depressive symptoms in our group of marginalized women are generalizable to low-income women of color in California. Although more study participants were below the federal poverty level (46.8%) compared to county (20.1%) and state-wide (40.0%) estimates and a higher proportion did not complete high school (25.5% vs. 8.9% in San Francisco County and 16.3% in California), the higher prevalence of adverse socio-economic conditions is related to over-representation of Latino mothers served by the safety-net hospital [43, 44]. An additional strength of our study includes the examination of a wide array of covariates, many objectively obtained from medical records. Moreover, all participants delivered in the same birth setting, thereby minimizing variation in health care service delivery.

Our study faced several limitations. Although we recognize bi-directionality, we only assessed the effect of stressors on gestational age but not the effect of complications of pregnancy such as preterm labor or preterm delivery on emotional distress. In fact, we lacked data on other psychosocial risks such as maternal anxiety or perceived stress resulting from exposure to adverse social determinants, which may increase risk of PTB [46]. Second, the quantitative data did not allow us to explore the temporality of experiences of depression symptoms, social determinants of health, and receipt of behavioral health services during pregnancy. Third we collected the data in the third trimester or retrospectively in the postpartum period, putting our study at risk for recall bias. Fourth, the small quantitative sample size resulted in statistical imprecision. The outliers retained in gestational age resulted in large confidence intervals. Further, we were limited in our capacity to explore stratified analyses to examine whether there were differential associations by race and/or ethnicity. The sample size also limited our ability to determine the effect modification of behavioral health services on the relationships between mental health symptoms, social determinants, and gestational age, for specific at-risk groups. Also, depression symptoms were measured with a single item which may not have the sensitivity and specificity of a validated prenatal depression screen. Behavioral health providers were also limited to social workers and counselors. Further, although all participants were eligible for a nurse home visiting program, we did not control for receipt of other services or interventions that participants might have opted to receive such as group prenatal care, Women, Infants, and Children (WIC), or prenatal care in a clinic. Any of these programs may have provided wraparound care comparable to behavioral health services, which might have influenced both exposures and the outcome. Additionally, we did not control for the use of anti-depressants, alcohol consumption, or smoking, which may be important confounders in the relationship between depression and PTB. Finally, ours was a convenience sample that may have introduced bias; women experiencing more stressors or more severe depression symptoms may have been less likely to participate, or may have had missing data on depression symptoms or treatment. Thus our findings may not be generalizable to all women at risk for preterm delivery. Nonetheless, we hypothesize that with a representative sample, we would have measured an even stronger relationship between key exposures and gestational age.

### Recommendations for research

Our findings indicate a number of areas for future research. A larger prospective quantitative study must be designed that includes more specificity around the timing and severity of mental health symptoms and their contributing factors (i.e. biological or contextual) [47], the types and/or combinations of specific social determinants that place women at highest risk, and behavioral health services that might mitigate those risks during pregnancy. Accurate measurement of the timing of the exposures would allow us to explore the pathways between mental health, social determinants, and birth outcomes, and any associated mediators or moderators, such as stress or health behaviors. Such studies should be adequately powered and utilize a sampling strategy that ensures participants are representative of the heterogeneity of the population, to allow for stratification of data by race and ethnicity. Future research would benefit from a matched sample of low risk populations as a control or comparison group to measure associations between depression symptoms, social determinants of health, and behavioral health services and gestational age among marginalized women compared to the general population.

With additional research, if exposure to adverse social determinants of health and presence of mental health symptoms are found to be uniquely and/or jointly associated with gestational age, and behavioral health services is corroborated to mitigate these risks, interventions can be developed to reduce the risk of preterm birth. The broader PTBR project, focusing on the creation of a centralized database of risk and protective factors for preterm birth, was designed in order to foster a rapid-cycle research to practice model. Further research is needed on the effectiveness of this approach, particularly the creation of a repository of risk factors including social determinants of health and mental health, and the use of such data to inform clinical practice. Finally, this study, if supported by further research, can also inform an intervention research study focusing on the development, implementation, and evaluation of behavioral health services during pregnancy. This research should be grounded in a strengths-based approach, focusing on mechanisms that support resilience and reduction of adverse birth outcomes among pregnant women [48].

## Conclusion

Building on a small sample of marginalized women of color at risk for preterm delivery, this study provides significant evidence that depressive symptoms during pregnancy and exposure to a high number of social determinants co-occurring with depressive symptoms are associated with shorter gestational ages at birth, even when controlling for other psychosocial factors. In addition, the findings show a possible protective effect of behavioral health services that might help to buffer the effect of depression symptoms and multiple adverse social determinants. Integrating obstetric and behavioral health services might enhance patient centered care for high-risk pregnant women.

## Supporting information

S1 Table Social determinant domains and distribution across items for each domain (n = 47). (PDF)

S1 File Minimal dataset containing prenatal depression, adverse social determinants, and gestational age measures (n = 47). (DTA)

S2 File File containing data collection instruments in English and Spanish. (PDF)
