# Supplementary material for: Preterm birth and social support services for prenatal depression and social determinants
Source: PLoS One. 2021 Aug 13;16(8):e0255810. doi: 10.1371/journal.pone.0255810 (PMC8362957; doi:10.1371/journal.pone.0255810)
Supplement: S1 Table — (PDF) [file pone.0255810.s001.pdf]

Domain	n	%
Work		
I missed a prenatal appointment because I was worried about losing my job	23	48.9
I missed a prenatal appointment because I couldn't take off work	13	27.7
I work overtime	13	27.7
During my pregnancy, I was regularly standing for more than 3 hours a day	11	23.4
During my pregnancy, I was lifting or carrying more than 25 pounds multiple times per hour	10	21.3
During my pregnancy, I was working a night shift or overnight shift at least once per week	9	19.1
During my pregnancy, I was bending or stooping multiple times per hour	8	17.0
During my pregnancy, I experienced a workplace with smoke or exhaust fumes every day	8	17.0
During my pregnancy, I was using chemicals like cleaning products or salon products every day	7	14.9
I requested changing some of my work activities but my employer/boss said no	7	14.9
No, I didn't request any changes to my work activities because I didn't know that I could	3	6.4
I took unpaid leave from my job	3	6.4
During my pregnancy, I lost my job even though I wanted to go on working	3	6.4
I was afraid I'd lose my job if I took leave	2	4.3
I had too much work to do to take leave	1	2.1
My job does not have paid leave	1	2.1
I had not built up enough leave time to take time off	0	0.0
Income		
It was very hard or somewhat hard for me and my family to live on my income	23	48.9
During my pregnancy, I had a lot of bills I could not pay	12	25.5
During my pregnancy, my partner lost their job	5	10.6
I could not financially afford to take leave	5	10.6
I missed a prenatal appointment because I had problems with my health insurance or I didn't have enough money to pay for my visits	3	6.4
During my pregnancy, my partner or I had our pay or hours cut back	2	4.3
Food Insecurity		
My family and I worried about having enough food	2	34.0
Housing Instability		
The place I lived for most of the pregnancy did not have a private bathroom	12	25.5
I had to move to a new address during pregnancy	11	23.4
