# Supplementary material for: Preterm birth and social support services for prenatal depression and social determinants
Source: PLoS One. 2021 Aug 13;16(8):e0255810. doi: 10.1371/journal.pone.0255810 (PMC8362957; doi:10.1371/journal.pone.0255810)
Supplement: S2 File — (PDF) [file pone.0255810.s003.pdf]

Survey

Please complete the survey below.

Thank you!

---

1) Study identifier across all instruments ________________________

Preterm Birth Review Study 2017
PIs: R. Jackson, J. Stookey

Consent Information

2) Date HIPAA Consent Signed ________________________

3) Date PTBR consent signed ________________________

The following questions are about the time just before you got pregnant with your baby who was just born.

As a reminder, you can skip any questions or stop taking the survey at any point. If you have any questions during the survey, please ask us for help.

4) During the month before you got pregnant, did you have Medi-Cal, private insurance, or some other health insurance plan for your own health care, or were you uninsured?
- ◯ Medi-Cal or a health plan paid for by Medi-Cal like San Francisco Health Plan
- ◯ Private Insurance
- ◯ Healthy San Francisco
- ◯ Other
- ◯ I did not have Medi-Cal or any other health insurance during the month before I got pregnant

5) Just before you got pregnant, did you have a particular doctor, nurse, or clinic that you usually went to if you wanted health care?
- ◯ Yes
- ◯ No

6) Before you got pregnant, would you say that your health was usually
- ◯ Excellent
- ◯ Very Good
- ◯ Good
- ◯ Fair
- ◯ Poor

7) Did you have any of the following before your pregnancy?
- ☐ High Blood Pressure
- ☐ Diabetes
- ☐ Asthma
- ☐ Depression or Anxiety
- ☐ None of the above

8) How many weeks pregnant were you when you were sure you were pregnant? For example, you had a pregnancy test or a doctor or nurse said you were pregnant. ________________________ (Weeks)

9) Did you receive treatment from a doctor, nurse, or other health care worker to help you get pregnant with this baby? For example, infertility treatments such as fertility-enhancing drugs or assisted reproductive technology.

◯ Yes
◯ No

10) What form of birth control were you using when you got pregnant? (Check all that apply)

If you were not using birth control, check only the last option.

☐ Condom
☐ IUD
☐ Depo provera
☐ Implant
☐ OCP
☐ Nuva Ring
☐ Patch
☐ Withdrawal
☐ I was not using birth control

11) Thinking back to just before you got pregnant, how did you feel about getting pregnant?

◯ I wanted to get pregnant then
◯ I wanted to get pregnant later
◯ I didn't want to get pregnant then or in the future
◯ I wasn't sure what I wanted

12) When you got pregnant, did you have a partner that lived with you?

◯ Yes, I had a partner AND they lived with me
◯ No, I had a partner BUT they did NOT live with me
◯ No, I did NOT HAVE a partner

13) Before you got pregnant, did you typically get a period every month?

◯ Yes
◯ No

14) If you have had a previous birth, when was your last birth?

If you have not given birth prior to this baby, please put "0".

____
(Months)

The next questions are about any medical care you might have received during your pregnancy.

Remember, you can skip questions or stop taking the survey at any time. You can also skip questions if they do not apply to your experience.

15) About how many weeks pregnant were you when you first tried to make an appointment to get prenatal care?

____
(Weeks)

16) Did you have group prenatal care or Centering Pregnancy?

◯ Yes
◯ No

17) During your pregnancy, did you do any of these things? (check all that apply)

☐ Had 'high risk' or specialty prenatal care
☐ Was hospitalized for one or more nights for pre-term labor
☐ Went to Labor & Delivery unit because I thought I might have preterm labor
☐ Doctor sewed my cervix closed (cerclage)
☐ Had bed rest for one or more weeks at home
☐ None of the Above

18) Thinking about the place you got the majority of your prenatal care, how did you typically get to prenatal care appointments?

- ◯ Walk
- ◯ Bus/public transit
- ◯ Taxi/Uber
- ◯ Drive self
- ◯ Get a ride with someone else
- ◯ Other

19) How long did it take to travel (one way) to receive prenatal care?

- ◯ Less than 20 minutes
- ◯ 20-40 minutes
- ◯ 40 minutes to 1 hour
- ◯ 1 hour to 1 ½ hours
- ◯ More than 1 ½ hours

20) There are many appointments for prenatal care during pregancy and it can be hard to attend all of them. Did any of the following make you miss an appointment or prevent you from receiving pre-natal care? (check all that apply)

If you didn't miss any appointments, check only the last option.

- ☐ I was worried about losing my job
- ☐ There was no one to take care of my children
- ☐ I had no way to get to the clinic or doctor's office
- ☐ I couldn't take time off work
- ☐ I had problems with my health insurance or I didn't have enough money to pay for my visits
- ☐ I had problems making an appointment
- ☐ I didn't think it was necessary to have so many appointments
- ☐ I didn't know where to go for prenatal care
- ☐ I didn't want anyone to know I was pregnant
- ☐ I didn't know I was pregnant
- ☐ I couldn't find a doctor or nurse who spoke my langauge
- ☐ I didn't miss any of my prenatal care appoinments

21) We would like to know how you felt about the prenatal care you received during this pregnancy. Please check all the parts of prenatal care that you were HAPPY with. (check all that apply)

- ☐ The amount of time I had to wait after I arrived for my visits
- ☐ The amount of time the doctor, nurse or midwife spent with me during my visits
- ☐ The advice I got on how to take care of myself
- ☐ The understanding and respect that the staff showed toward me as a person
- ☐ Services available like nutrition, nursing, social work
- ☐ Classes and education
- ☐ None of the above

22) In general, do you trust your prenatal care providers?

- ◯ Always
- ◯ Most of the time
- ◯ Sometimes
- ◯ Rarely/Never

23) Overall, how happy were you with the prenatal care you received?

- ◯ Not at all satisfied
- ◯ Somewhat dissatisfied
- ◯ No opinion/Neutral
- ◯ Somewhat satisfied
- ◯ Satisfied
- ◯ Not applicable

24) In general, do you trust that the health care system will meet your needs?

- ◯ Always
- ◯ Most of the time
- ◯ Sometimes
- ◯ Rarely/Never

The next questions are about your work experiences during your pregnancy.

Remember, you can skip questions or stop taking the survey at any time. You can also skip questions if they do not apply to your experience.

25) During your pregnancy, what type of work did you do?

____
(Ex: waiter, nurse, cashier, lawyer, unemployed)

---

26) When during your pregnancy did you work at a job for pay? (check all that apply)

If you didn't work for pay during your pregnancy, check only the last option.

☐ 1st trimester (0-3 months of pregnancy)
☐ 2nd trimester (3-6 months of pregnancy)
☐ 3rd trimester (6-9 months of pregnancy)
☐ I did not work at a job for pay during my pregnancy

---

27) When did you stop working?

____
((enter a date MM-DD-YYYY))

---

28) During your pregnancy, how many hours did you work per week at your job or jobs?

◯ 50+ hours per week
◯ 40-49 hours per week
◯ 30-39 hours per week
◯ 20-29 hours per week
◯ 10-19 hours per week
◯ Less than 10 hours per week
◯ I did not work

---

29) During your pregnancy, did you experience any of the following things? (check all that apply)

☐ Regularly standing for more than 3 hours a day
☐ Lifting or carrying more than 25 pound multiple times per hour
☐ Working a night shift or overnight shift at least once a week
☐ Bending or stooping multiple times per hour
☐ Workplace with smoke or exhause fumes every day
☐ Using chemicals like cleaning products or salon products every day
☐ None of the above

---

30) Some people are able to change their job activities to help them continue to work while pregnant. Was this something you tried to do?

◯ Yes, I was able to change some of my work activities
◯ I requested this but my employer/boss said no
◯ No, I didn't request any changes because I didn't know that I could
◯ No, I didn't request any changes because I didn't feel I needed them
◯ None of the above

---

31) Which of the following describes the leave or time you took off from work before this baby was born? (check all that apply)

☐ I took paid leave from my job and my employer paid me
☐ I took paid leave from my job and the state disability program paid me
☐ I took unpaid leave from my job
☐ I did not take leave

---

32) Here are a few things that might happen to some people during their pregnancies. Did any of these things happen to you during this pregnancy?

☐ My partner lost their job
☐ I lost my job even though I wanted to go on working
☐ My partner or I had our pay or hours cut back
☐ I had a lot of bills I could not pay
☐ None of the Above

33) Did any of the things listed below affect your decision about taking leave from work before this baby was born? (check all that apply)

- ☐ I could not financially afford to take leave
- ☐ I was afraid I'd lose my job if I took leave
- ☐ I had too much work to do to take leave
- ☐ My job does not have paid leave
- ☐ I had not built up enough leave time to take time off
- ☐ I did not take leave because I did not want to
- ☐ I did not take leave because I gave birth prior to the start of my planned leave
- ☐ None of the above

The next questions are about your health during your pregnancy.

Remember, you can skip questions or stop taking the survey at any time. You can also skip questions if they do not apply to your experience.

34) Overall, how was this pregnancy experience?

- ◯ One of the happiest times of my life
- ◯ A happy time with few problems
- ◯ A moderately hard time
- ◯ A very hard time
- ◯ One of the worst times of my life

35) During your pregnancy, would you say that your health was usually:

- ◯ Excellent
- ◯ Very Good
- ◯ Good
- ◯ Fair
- ◯ Poor

36) Did you douche at any time during your pregnancy (for example, using products such as Summer's Eve, Massengill, or vinegar/water solution)?

- ◯ Yes
- ◯ No

37) How long has it been since you last visited a dentist or a dental clinic for any reason? Include visits to dental specialists, such as orthodontists.

- ◯ Within the past year (anytime less than 12 months ago)
- ◯ Within the past 2 years (1 year but less than 2 years ago)
- ◯ Within the past 5 years (2 years but less than 5 years go)
- ◯ I've never been to the dentist

38) During your pregnancy, did you have problems with your teeth or gums?

- ◯ Yes
- ◯ No

39) Were you able to see a dentist or dental clinic about your dental problem?

- ◯ Yes
- ◯ No
- ◯ I DID NOT HAVE any dental problems

40) During your pregnancy, did you do any of these things? (check all that apply)

- ☐ Took progesterone to prevent labor
- ☐ Took other medicine to prevent labor
- ☐ Took prescribed medicine to control blood pressure
- ☐ Took prescribed medicine to control blood sugar
- ☐ Took aspirin
- ☐ None of the above

41) During the last 3 months of this pregnancy, how often did you participate in any physical activities or exercise for 30 minutes or more? (For example, walking for exercise, swimming, cycling, dancing, or gardening.) Do not count exercise you may have done as part of your regular job.

- ◯ I didn't exercise
- ◯ I didn't exercise, because was told by a doctor, nurse, or health care worker not to exercise
- ◯ Less than 1 day per week
- ◯ 1 to 4 days per week
- ◯ 5 or more days per week

42) During your pregnancy, how often did you eat something that you bought ready-to-eat? (like rotisserie chicken, sandwich, or pre-packaged salad)

- ◯ Everyday
- ◯ Multiple times during the week
- ◯ Sometimes (A few times each month)
- ◯ Rarely (Less than once per month)
- ◯ Never (I cook all meals from raw ingredients)

43) During your pregnancy, what kind(s) of water did you drink? (check all that apply)

- ☐ Tap water from the sink at home
- ☐ Tap water from a water fountain at work
- ☐ Water from tap stations in public places (like at parks, schools)
- ☐ Bottled water or water from a restaurant or store
- ☐ I didn't usually drink plain water

44) During your pregnancy, how many glasses of water did you drink at school/work, home and everywhere else per day?

(Count one cup as one glass and count one bottle of water as two glasses. Count only a few sips, like from a water fountain, as less than one glass.)

____

45) How often does anyone smoke inside your home?

- ◯ Daily
- ◯ Weekly
- ◯ Monthly
- ◯ Less than monthly
- ◯ Never
- ◯ Don't know

46) During your pregnancy, how often was the following statement true?

"My family and I worried whether our food would run out before we got money to buy more."

- ◯ Often true
- ◯ Sometimes true
- ◯ Never true

47) During your pregnancy, did you ever have 2 weeks or longer when you felt sad, empty, or depressed for most of the day?

- ◯ Yes
- ◯ No

The next questions are about life experiences during your pregnancy.

Remember, you can skip questions or stop taking the survey at any time. You can also skip questions if they do not apply to your experience.

48) Here are a few things that might happen to some people during their pregnancies. Did any of these things happen to you during this pregnancy?

- ☐ I got separated or divorced from my spouse or partner
- ☐ My partner or I went to jail
- ☐ Someone very close to me had a problem with drinking or drugs
- ☐ A close family member was very sick and had to go into the hospital
- ☐ I argued with my spouse or partner more than usual.
- ☐ I was in a physical fight
- ☐ None of the Above

49) During your pregnancy, how much were you worried or upset about experiencing unfair treatment or discrimination because of your race, ethnicity, gender, religion, sexual orientation, or citizenship status?

- ◯ Most of the time
- ◯ Some of the time
- ◯ Rarely or Never

The following question is about difficult experiences some people have with their intimate partner.

Remember, you can skip questions or stop taking the survey at any time. You can also skip questions if they do not apply to your experience.

50) During your pregnancy, did you partner threaten or physically hurt you?

- ◯ Yes
- ◯ No
- ◯ I did not have a partner

The next questions are about difficult childhood experiences that some people might experience.

Remember, you can skip questions or stop taking the survey at any time. You can also skip questions if they do not apply to your experience.

51) Thinking back to your childhood through age 18, how often was it hard for your family to pay for basic needs like food or housing?

- ◯ Very often
- ◯ Somewhat often
- ◯ Not very often
- ◯ Never

52) Before your 18th birthday, did you experience verbal, physical, emotional and/or sexual abuse?

- ◯ Yes
- ◯ No

The next questions are about the types of support and community you had during your pregnancy.

Remember, you can skip questions or stop taking the survey at any time. You can also skip questions if they do not apply to your experience.

53) During this pregnancy, did you receive any of the following services or information? (check all that apply)

- ☐ A class or classes to prepare for childbirth or parenting
- ☐ A home visit to help prepare for the new baby
- ☐ Breastfeeding information or class
- ☐ Counseling for personal or family problems, stress, depression or anxiety
- ☐ Help from a social worker in clinic
- ☐ Help with an alcohol or drug problem
- ☐ Help to reduce violence in my home
- ☐ Help to quit smoking
- ☐ None of the above

54) During your pregnancy, did you have someone to help you with daily tasks, like getting a ride somewhere, shopping, or cooking?

- ◯ Yes
- ◯ No

55) During this pregnancy, did you receive any of the following services or information? (check all that apply)

- ☐ Legal Aid (help from a lawyer)
- ☐ Financial help or education
- ☐ Pregnancy or parenting Apps (applications) for my cellphone or computer
- ☐ Residential treatment
- ☐ Food from food pantry or church
- ☐ Financial help or a loan from family, friend, church
- ☐ None of the above

56) During your pregnancy, did you have someone you could turn to if you needed someone to comfort you or listen to you?

- ◯ Yes
- ◯ No

57) During this pregnancy, did you receive any of the following services or information? (check all that apply)

- ☐ Food stamps, CalFresh benefits, or money to buy food,
- ☐ WIC benefits (Women Infant Children Nutrition Program)
- ☐ CalWorks or welfare
- ☐ Services from Black Infant Health
- ☐ Services from HPP-Homeless Prenatal Partnership
- ☐ None of the above

58) Do you consider yourself a religious or spiritual person?

- ◯ Very Much
- ◯ Moderately
- ◯ Slightly
- ◯ Not at all

59) During pregnancy, how satisfied were you with the support given by your baby's father?

- ◯ Not at all satisfied
- ◯ Somewhat dissatisfied
- ◯ Somewhat satisfied
- ◯ Very satisfied
- ◯ Not applicable

This is the last section of questions. It focuses on demographic questions.

Remember, you can skip questions or stop taking the survey at any time. You can also skip questions if they do not apply to your experience.

60) Do you consider yourself Hispanic / Latinx?

- ◯ Yes
- ◯ No

61) What categories best describe you? Select all that apply.

- ☐ Black or African American
- ☐ Chinese
- ☐ Filipino
- ☐ Other Asian
- ☐ Native American (American Indian/Alaska Native)
- ☐ Native Hawaiian or other Pacific Islander
- ☐ White
- ☐ Other

62) If you chose "other" in the above question, please specify:

____

63) What countries or regions best describe your racial/ethnic background? Please write in any that apply.

For example, Chinese, Russian, Chilean.

____

64) During your pregnancy, what neighborhood, zip code did you live in? (Please check all the neighborhoods where you lived)

- ☐ I did not live in any of these areas
- ☐ Bayview, Hunters Point, 94124
- ☐ Chinatown, 94108
- ☐ Castro, Noe Valley, Corona Heights, 94114
- ☐ Excelsior, Ocean View, 94112
- ☐ Haight Ashbury, Hayes Valley, 94117
- ☐ Inner Richmond, Presidio, 94118
- ☐ Lake Merced, Lake Shore, 94132
- ☐ Marina, Cow Hollow, 94123
- ☐ Mission, Bernal Heights, 94110
- ☐ Nob Hill, Russian Hill, 94109
- ☐ North Beach, Telegraph Hill, 94133
- ☐ Parkside, Forest Hill, 94116
- ☐ Potrero Hill, Financial District, 94107
- ☐ Presidio, 94129
- ☐ Richmond, Sea Cliff, 94121
- ☐ Rincon Hill, 94105
- ☐ South of Market, 94103
- ☐ St Francis Wood, West Portal, 94127
- ☐ Sunset, 94122
- ☐ Tenderloin, Hayes Valley, 94102
- ☐ Twin Peaks, Diamond Heights, Glen Park, 94131
- ☐ Visitacion Valley, Portola, 94134
- ☐ Western Addition, Pacific Heights, 94115

65) How safe from crime do you consider your neighborhood to be?

- ◯ Very safe
- ◯ Somewhat safe
- ◯ Somewhat unsafe
- ◯ Not at all safe
- ◯ Don't know

66) What kind of place did you live in for most of the pregnancy?

- ◯ Apartment, flat
- ◯ Trailer, mobile home
- ◯ Public Housing, Section 8
- ◯ With friends (on couch or floor)
- ◯ Shelter, boarding house
- ◯ Car, Van
- ◯ On the street or in a tent
- ◯ House, townhouse, condo
- ◯ SRO, Motel, or Hotel
- ◯ None of the Above

67) Not counting you, how many people lived with you that were 18 years old or older?

- ◯ 5+ people
- ◯ 3-4 people
- ◯ 1-2 people
- ◯ 0 people

68) Not counting you, how many people lived with you that were 17 years old or younger?

- ◯ 5+ people
- ◯ 3-4 people
- ◯ 1-2 people
- ◯ 0 people

69) Did the place you live have any of the following? (check all that apply)

- ☐ Full Kitchen
- ☐ Private Bathroom
- ☐ None of the above

70) Here are a few things that might happen to some people during their pregnancies. Please tell us if any of these things happened to you during this pregnancy:

- ☐ I had to move to a new address
- ☐ I did not have a regular place to sleep at night (had to move from house to house)
- ☐ I was homeless (had to sleep outside, or stay in a car or a shelter)
- ☐ None of the Above

71) How long have you lived in the United States?

- ◯ Less than 5 years
- ◯ 5-10 years
- ◯ 10-15 years
- ◯ 15-20 years
- ◯ 20+ years
- ◯ Entire Life

72) Do you feel like you have a strong cultural community that can help you find services and resources in the Bay area?

- ◯ Yes, all the time
- ◯ Yes, some of the time
- ◯ No, rarely or never

73) What was your total family income before taxes this past year?

Please mark one box below that includes your total family income, including your income and the income of your spouse or partner (if living with you during this time) and your children.
Please include income from all sources, including jobs, welfare, Disability, Unemployment, child support, interest, dividends, and support from family members.

- ◯ $0 to $16,000
- ◯ $16,001 to $20,000
- ◯ $20,001 to $24,000
- ◯ $24,001 to $28,000
- ◯ $28,001 to $31,000
- ◯ $31,001 to $36,000
- ◯ $36,001 to $39,000
- ◯ $39,001 to $47,000
- ◯ $47,001 to $55,000
- ◯ $55,001 to $59,000
- ◯ $59,001 to $63,000
- ◯ $63,001 to $71,000
- ◯ $71,001 to $78,000
- ◯ $78,001 to $83,000
- ◯ $83,001 to $94,000
- ◯ $94,001 to $107,000
- ◯ $107,001 to $110,000
- ◯ $110,001 to $126,000
- ◯ $126,001 to $142,000
- ◯ $142,001 or more

---

74) How many people lived on your household income?

____
(Type a number (ex: 3))

---

75) In general, during this pregnancy, how hard was it for you and your family to live on the income you had?

- ◯ Very hard
- ◯ Somewhat hard
- ◯ Not too hard
- ◯ Not hard at all

---

76) What language do you usually speak at home?

- ◯ English
- ◯ Spanish
- ◯ English and Spanish equally
- ◯ Cantanese
- ◯ Russian
- ◯ Vietnamese
- ◯ Ukranian
- ◯ Arabic
- ◯ None of the Above

---

77) How confident are you filling out medical forms by yourself?

- ◯ Extremely
- ◯ Moderately
- ◯ A little bit
- ◯ Not at all

You are now finished with the survey.

Thank you so much for taking the time to fill out the survey! You have contributed positively to helping future parents and children.

Please check-in with the researcher now, and they will explain the next steps.

---

78) I am finished with the survey.

- ◯ Yes

Survey

Please complete the survey below.

Thank you!

---

1) Study identifier across all instruments

____

Preterm Birth Review Study 2017
PIs: R. Jackson, J. Stookey

Informacion sobre consentimiento

2) Fecha HIPAA consentimiento firma

____

3) Fecha PTBR consentimiento firma

____

Las siguientes preguntas son acerca del tiempo justo antes de quedar embarazada con este embarazo.

Recuerde que usted puede saltar cualquier pregunta o parar de hacer la encuesta en cualquier momento. Usted también puede saltar preguntas si no aplican con su propia experiencia.

Si tiene alguna pregunta durante la encuesta, por favor pídanos ayuda.

4) Durante el mes antes de quedar embarazada, ¿tenía usted cobertura de Medi-Cal, seguro médico privado, u otro plan de salud para su propia atención médica, o no tenía seguro?

- ◯ Medi-Cal o un plan de salud pagado por Medi-Cal
- ◯ Un seguro médico privado
- ◯ Healthy San Francisco
- ◯ Otro
- ◯ Yo no tenía Medi-Cal ni otro seguro de salud durante el mes antes del comienzo de mi embarazo

5) Poco antes de quedar embarazada, ¿tenía usted un doctor, enfermera o clínica adonde generalmente iba cuando quería recibir atención médica?

- ◯ Sí
- ◯ No

6) Antes de quedar embarazada, diría usted que, en general, su salud era-

- ◯ Excelente
- ◯ Muy buena
- ◯ Buena
- ◯ Regular
- ◯ Mala

7) Tuvo alguno de los siguiente antes de su embarazo?

- ☐ Presión alta
- ☐ Diabetes
- ☐ Asma
- ☐ Depresión o Ansiedad
- ☐ Ninguna de las opciones anteriores

8) ¿Cuántas semanas pde embarazo tenía usted cuando estuvo segura de que estaba embarazada? Por ejemplo, se hizo una prueba de embarazo o un doctor o enfermera le dijo que estaba embarazada.

____
(Semanas)

9) ¿Recibió tratamiento de un médico, enfermera u otra trabajadora de la salud para ayudarle a quedar embarazada con este bebé? Por ejemplo, tratamientos para la infertilidad como fármacos para mejorar la fertilidad o tecnología de reproducción asistida.

- ◯ Sí
- ◯ No

10) ¿Qué método de anticonceptivo estaba usando cuando quedó embarazada? (Marque todos los métodos que apliquen.)

Si no estaba usando metodo de anticonceptivo, marque solo la ultima opción.

- ☐ Condón
- ☐ DIU
- ☐ Inyeccion Depo Provera
- ☐ Implante
- ☐ Pastillas
- ☐ Anillo
- ☐ Parche
- ☐ Retiro
- ☐ No estava usando un metodo de anticonceptivo

11) Piense en el momento poco antes de quedar embarazada,¿cómo se sentía al respecto a quedar embarazada?

- ◯ Quería quedar embarazada en ese momento
- ◯ Quería quedar embarazada más adelante
- ◯ No quería quedar embarazada ni en ese momento ni en el futuro
- ◯ No estaba segura de lo que quería

12) Cuando usted quedó embarazada, ¿usted tenía una pareja viviendo con usted?

- ◯ Si, tenía una pareja Y vivía conmigo
- ◯ No, tenía una pareja PERO NO vivía conmigo
- ◯ No, yo NO TENIA una pareja

13) Antes de quedar embarazada, ¿por lo regular, usted mentruaba cada mes?

- ◯ Sí
- ◯ No

14) Si ha tenido un nacimiento en el pasado, ¿cuántos meses atrás fue su último nacimiento?

Por favor escriba "0" si no ha tenido un nacimiento antes.

____
(Meses)

Las sigientes preguntas son sobre cualquier cuidado médico que usted ha recibido durante su embarazo.

Recuerde que usted puede saltar cualquier pregunta o parar de hacer la encuesta en cualquier momento. Usted también puede saltar preguntas si no aplican con su propia experiencia.

15) ¿Como cuántas semanas embarazada estaba cuando primero trato de hacer una cita para el cuidado prenatal?

____
(Semanas)

16) ¿Usted tuvo cuidado prenatal en grupo o de Centering Pregnancy?

- ◯ Sí
- ◯ No

17) Durante su embarazo, ¿hizo algunas de usta cosas? (Marque todas que apliquen.)

- ☐ Tuvo cuidado prenatal de "alto riesgo" o especializado
- ☐ Estuvo hospitalizada por una o dos noches por trabajo de parto prematuro
- ☐ Fue al departamento de parto porque pensó que estaba teniendo un parto prematuro
- ☐ El doctor cerró el cuello de la matriz
- ☐ Tuvo que estar encamada por una o dos semanas en casa
- ☐ Ninguna de las opciones anteriores

18) Pensando en el lugar en donde recibió la mayoría de su cuidado prenatal, ¿por lo regular cómo llegaba a sus citas prenatales?

- ◯ Caminando
- ◯ Camión/ Tránsito público
- ◯ Taxi/Uber
- ◯ Manejé
- ◯ Una persona me dio un ride
- ◯ Otro modo

19) ¿Cuánto tiempo tomó para viajar (solo de ida) para recibir cuidado prenatal?

- ◯ Menos de 20 minutos
- ◯ 20-40 minutos
- ◯ 40 minutos a una hora
- ◯ 1 hora a una hora y media
- ◯ Más de una hora y media

20) Hay muchas citas para cuidado prenatal durante el embarazo y puede ser difícil assistir a todas. Algunas de las sigientes cosas la hizo faltar a una cita o le impidió recibir cuidado prenatal? (Marque todas que apliquen.)

Si no faltó a una cita, marque solo la última respuesta.

- ☐ Estaba precupada perder mi trabajo
- ☐ No habia nadie para cuidar a mis niños
- ☐ No tenía modo de llegar a la clínica o consultorio
- ☐ No pude tomar tiempo del trabajo
- ☐ Tuve problema con mi seguro de salud o no tenía dinero para pagar la consulta
- ☐ Tenía probemas haciendo una cita
- ☐ No pensaba que era necesario tener tantas citas
- ☐ No sabía a donde acudir para citas de cuidado prenatal
- ☐ No quería que nadie se diera cuenta que estaba embarazada
- ☐ No sabía que estaba embarazada
- ☐ No podía encontrar un doctor o enfermera que hablara mi idioma
- ☐ No falté a ninguna cita de cuidado prenatal

21) Nos gustaría saber como se sintió con su cuidado prenatal que recibío durante su embarazo. Favor de marcar todas las partes de su cuidado prenatal con las que estuvo FELIZ (Marque todas que apliquen.)

- ☐ La cantidad de tiempo que tuvo que esperar después de llegar para mi visita
- ☐ La cantidad de tiempo el doctor, enfermera o partera tuvo conmigo durante mi visita
- ☐ Los consejos que me dieron sobre como cuidarme a mi misma
- ☐ El entendimiento y respeto que el personal mostró hacia mí como una persona
- ☐ Servicios desponibles como de nutrición, enfermería y trabajadora social
- ☐ Clases y educación
- ☐ Ninguna de las opciones anteriores

22) En general, ¿usted confía en su proveedor de cuidado prenatal?

- ◯ Siempre
- ◯ La mayoría del tiempo
- ◯ A veces
- ◯ Raramente/Nunca

23) Sobre todo, ¿qué tan feliz estuvo usted con el cuidado prenatal que recibió?

- ◯ De ningún modo satisfecha
- ◯ Algo insatisfecha
- ◯ No tengo opinión/neutral
- ◯ Algo satisfecha
- ◯ Satisfecha
- ◯ No aplica

24) En general, ¿usted confía que el sistema de cuidado de salud satisfará sus necesidades?

- ◯ Siempre
- ◯ La mayoría del tiempo
- ◯ A veces
- ◯ Raramente/Nunca

Las sigientes preguntas son sobre su experiencia durante su embarazo.

Recuerde que usted puede saltar cualquier pregunta o parar de hacer la encuesta en cualquier momento. Usted también puede saltar preguntas si no aplican con su propia experiencia.

25) Durante su embarazo, ¿qué tipo de trabajo hizo?

____
(Por ejemplo: mesera, enfermera, cajera, abogada, sin empleo)

26) ¿Hasta cuándo trabajó en un trabajo por paga durante su embarazo? (Marque todas que apliquen).

Si usted no trabajó por paga durante su embarazo, solamente marque la última opción.

- ☐ Primer trimestre (0-3 meses de embarazo)
- ☐ Segundo trimestre (3-6 meses de embarazo)
- ☐ Tercer trimestre (6-9 meses de embarazo)
- ☐ No trabajé en un trabajo que paga durante el embarazo

27) ¿Cuándo paró de trabajar?

____
(Escriba la fecha MM-DD-AAAA)

28) Durante su embarazo más reciente, ¿cuántas horas trabajó por semana en su empleo principal?

- ◯ 50 horas o más por semana
- ◯ 40 a 49 horas por semana
- ◯ 30 a 39 horas por semana
- ◯ 20 a 29 horas por semana
- ◯ 10 a 19 horas por semana
- ◯ Menos de 10 horas por semana
- ◯ No trabajé

29) Durante su embarazo, ¿Tuvo usted lo siguiente? (Marque todas que apliquen.)

- ☐ Estar parada regularmente por más de 3 horas al día
- ☐ Levantando o cargando más de 25 libras varias veces por hora
- ☐ Trabajando por la noche o turno de noche a lo menos una ves a la semana
- ☐ Doblar o agacharse varias veces por hora
- ☐ Trabajo con humo o gases de escape todos los días
- ☐ Usando químicas como limpiadores o productos de salón todos los días
- ☐ Ninguna de las opciones anteriores

30) Hay veces que mujeres pueden cambiar sus actividades de trabajo para ayudarlas continuar trabajando mientras estén embrazadas. ¿Esto era algo que usted trató de hacer?

- ◯ Sí, pude cambiar algunas de mis actividades del trabajo
- ◯ Solicité esto pero mi patrón/jefe digo que no
- ◯ No, no solicite ningún cambio porque no sabía que podía
- ◯ No, no solicité ningún cambio a mis actividades porque no sentí que lo necesitaba
- ◯ Ninguna de las opciones anteriores

31) ¿Cuáles de las siguientes opciones describe el tiempo de licencia que se tomó después del nacimiento de su nuevo bebé? (Marque todas que apliquen.)

- ☐ Me tomé licencia pagada
- ☐ Me tomé licencia pagada, y el programa de descapacidad del estado me pagó
- ☐ Me tomé licencia no pagada
- ☐ No me tomé licencia

32) Aquí son unas cosas que pueden pasarle a unas personas durante sus embarazos. ¿Algunas de estas cosas le pasó a usted durante su embarazo?

- ☐ Mi pareja perdió su trabajo
- ☐ Perdí mi trabajo aunque quería seguir trabajando
- ☐ Mi pareja o yo tuvimos nuestro cheque o nuestras horas de trabajo reducidas
- ☐ Tuve muchas cuentas que no pude pagar
- ☐ Ninguna de las opciones anteriores

33) ¿Alguna de las circunstancias que se enumeran aquí influyó en su decisión sobre tomar licencia después del nacimiento de su nuevo bebé?

- ☐ Económicamente no me fue posible tomar una licencia
- ☐ Tenía miedo de perder mi trabajo si tomaba licencia o si tomaba más tiempo
- ☐ Tenía demasiado trabajo pendiente para poder tomar licencia pagada o para tomar más tiempo
- ☐ Mi trabajo no tiene licencia pagada
- ☐ No había acumulado suficiente horas de licencia para tomar una licencia o para tomar más tiempo
- ☐ No tomé licencia porque yo no quería tomarla
- ☐ No tomé licencia porque di a luz antes del empiezo de mi licencia planeada.
- ☐ Ninguna de las opciones anteriores

Las sigientes preguntas son sobre su salud durante su embarazo.

Recuerde que usted puede saltar cualquier pregunta o parar de hacer la encuesta en cualquier momento. Usted también puede saltar preguntas si no aplican con su propia experiencia.

34) En general, ¿cómo fue su experiencia de embarazo?

- ◯ Unas de las etapas más feliz de mi vida
- ◯ Un tiempo feliz con pocos problemas
- ◯ Un tiempo algo difícil
- ◯ Un tiempo muy difícil
- ◯ Uno de las peores etapas de mi vida

35) Durante su embarazo, ¿diría usted que su salud era generalmente:

- ◯ Excelente
- ◯ Muy buena
- ◯ Buena
- ◯ Regular
- ◯ Mala

36) ¿Usted usó irrigación vaginal (la ducha) durante su embarazo?
Por ejemplo, usando productos como Summer's Eve, Massengill, o una solución de vinagre y agua.

- ◯ Sí
- ◯ No

37)	¿Cuánto tiempo ha pasado desde que visitó a un dentista o a una clínica dental por cualquier motivo? Incluya visitas a especialistas dentales, como ortodoncistas.	◯ En el último año (menos de 12 meses)<br>◯ En los último dos años (más de 1 año pero menos de 2 años)<br>◯ En los último 5 año (más de 2 años pero menos de 5 años)<br>◯ Nunca he ido al dentista
38)	Durante su embarazo, ¿tuvo problemas con los dientes o las encías?	◯ Sí<br>◯ No
39)	¿Fue capaz de ver a un dentista o a una clínica dental acerca de su problema dental?	◯ Sí<br>◯ No<br>◯ NO TENIA PROBLEMAS dentales
40)	Durante su embarazo, ¿hizo algunas de estas cosas? (Marque todas que apliquen.)	☐ Tomé progesterona para prevenir el embarazo<br>☐ Tomé otro medicamento para prevenir el embarazo<br>☐ Tomé medicamento para el control de la presión<br>☐ Tomé medicamento para controlar mi azucar<br>☐ Tomé aspirina<br>☐ Ninguna de las opciones anteriores
41)	Durante los últimos 3 meses de este embarazo, ¿con qué frecuencia participó en alguna actividad física o ejercicio durante 30 minutos o más? Por ejemplo, caminar para hacer ejercicio, nadar, andar en bicicleta, bailar o cultivar un jardín.<br><br>No cuente el ejercicio que puede haber hecho como parte de su trabajo regular.	◯ No hice ejercicio<br>◯ No hice ejercicio, porque el doctor, enfermera, o trabajadora de salud me dijo que no debo hacer ejercicio<br>◯ Menos de una vez a la semana<br>◯ 1 a 4 días por semana<br>◯ 5 o más días por semana
42)	Durante su embarazo, ¿con qué frecuencia comió algo que compró listo para comer? (Como pollo asado, sándwich, o ensalada pre-empaquetada)	◯ Todos los días<br>◯ Varias veces durante la semana<br>◯ A veces (unas veces al mes)<br>◯ Raramente (menos de una vez por semana)<br>◯ Nunca (Cocinó todas las comidas de ingredientes crudos)
43)	Durante su embarazo, ¿qué tipo(s) de agua bebió? (Marque todas que apliquen.)	☐ Agua del grifo del fregadero en casa<br>☐ Agua del grifo de una fuente de agua en el trabajo<br>☐ Agua de las estaciones del grifo en lugares públicos (como en parques, escuelas)<br>☐ Agua de botella o de un restaurante o tienda<br>☐ Usualmente no tomaba agua pura
44)	Durante su embarazo, ¿cuántos vasos de agua bebió en la escuela / trabajo, en casa y en todas partes cada día?<br><br>(Contar una taza como un vaso y contar una botella de agua como dos vasos. Cuente sólo unos tragos, como de una fuente de agua, como menos de un vaso.)	______________________
45)	¿Con qué frecuencia alguien fuma dentro de su casa?	◯ Todos los días<br>◯ Semanal<br>◯ Al mes<br>◯ Menos que mensual<br>◯ Nunca<br>◯ No se

46) Durante su embarazo, ¿con qué frecuencia fue la siguiente oración cierta?

"Mi familia y yo nos preocupamos si la comida se acabaría antes de que tuviéramos dinero para comprar más."

- ◯ A menudo es cierto
- ◯ A veces es cierto
- ◯ Nunca cierto

47) Durante su embarazo, ¿tuvo usted alguna vez un lapso de 2 semanas o más cuando se sentía triste, vacía o deprimida durante la mayor parte del día?

- ◯ Sí
- ◯ No

Las siguientes preguntas son acerca de experiencias de vida durante su embarazo.

Recuerde que usted puede saltar cualquier pregunta o parar de hacer la encuesta en cualquier momento. Usted también puede saltar preguntas si no aplican con su propia experiencia.

48) Aquí hay algunas cosas que pueden pasarle a algunas personas durante sus embarazos. ¿Alguna de estas cosas le sucedió durante este embarazo? (Marque todas que apliquen.)

- ☐ Me separé de mi pareja o nos divorciamos
- ☐ Mi pareja o yo estuvimos en la cárcel
- ☐ Alguien muy cerca de mí tenía un problema con el alcohol o las drogas
- ☐ Un familiar cercano estaba muy enfermo y tuvo que ir al hospital
- ☐ Discutí con mi cónyuge o pareja más de lo habitual
- ☐ Estaba en una pelea física
- ☐ Ninguna de las opciones anteriores

49) Durante su embarazo, ¿cuánto le preocupó o molestó tener la experiencia de un trato injusto o discriminación debido a su raza, etnia, género, religión, orientación sexual o estatus de ciudadanía?

- ◯ La mayoría del tiempo
- ◯ Algunas de las veces
- ◯ Rara vez o nunca

La siguiente pregunta es sobre experiencias difíciles que algunas personas tienen con su pareja(s) íntima(s).

Recuerde que usted puede saltar cualquier pregunta o parar de hacer la encuesta en cualquier momento. Usted también puede saltar preguntas si no aplican con su propia experiencia.

50) Durante su embarazo, ¿su pareja la amenazó o le hizo daño?

- ◯ Sí
- ◯ No
- ◯ No tenía pareja

Las siguientes preguntas son sobre las experiencias difíciles de la infancia que algunas personas podrían tener.

Recuerde que usted puede saltar cualquier pregunta o parar de hacer la encuesta en cualquier momento. Usted también puede saltar preguntas si no aplican con su propia experiencia.

51) Pensando en su niñez hasta la edad de 18 años, ¿con qué frecuencia fue difícil para su familia pagar las necesidades básicas, como comida y casa?

- ◯ Con mucha frecuencia
- ◯ Con algo de frecuencia
- ◯ Con poca frecuencia
- ◯ Nunca

52) Antes de cumplir 18 años, ¿tuvo abuso verbal, físico, emocional y / o sexual?

- ◯ Sí
- ◯ No

Las siguientes preguntas son acerca de los tipos de apoyo y comunidad que tuvo durante su embarazo.

Recuerde que usted puede saltar cualquier pregunta o parar de hacer la encuesta en cualquier momento. Usted también puede saltar preguntas si no aplican con su propia experiencia.

53) Durante este embarazo, ¿recibió alguno de los siguientes servicios o información? (Marque todas que apliquen.)

- ☐ Una clase(s) para preparar para parto o crianza
- ☐ Una visita en casa para ayudar preparar para el nuevo bebe
- ☐ Información o clase de lactancia materna
- ☐ Asesoramiento para problemas personales o familiares, estrés, depresión o ansiedad
- ☐ Ayuda de un trabajador social en la clínica
- ☐ Ayuda con un problema de alcohol o drogas
- ☐ Ayuda para reducir violencia en mi casa
- ☐ Ayuda para dejar de fumar
- ☐ Ninguna de las opciones anteriores

54) Durante su embarazo, ¿tenía usted alguien quien podía ayudarle con cosas cotidianas, como ir a algún sitio, hacer las compras, o cocinar?

- ◯ Sí
- ◯ No

55) Durante este embarazo, ¿recibió alguno de los siguientes servicios o información? (Marque todas que apliquen.)

- ☐ Ayuda Legal (ayuda de un abogado)
- ☐ Ayuda financiera o educación
- ☐ Aplicaciones en mi teléfono celular o computadora sobre el embarazo o la paternidad
- ☐ Tratamiento residencial
- ☐ Alimentos de la despensa de alimentos o la iglesia
- ☐ Ayuda financiera o un préstamo de la familia, amigo, iglesia
- ☐ Ninguna de las opciones anteriores

56) Durante su embarazo, ¿tenía alguien a quien podía recurrir para consolarla o escucharla?

- ◯ Sí
- ◯ No

57) Durante este embarazo, ¿recibió alguno de los siguientes servicios o información? (Marque todas que apliquen.)

- ☐ Cupones de alimentos, beneficios de CalFresh o dinero para comprar comida
- ☐ Beneficios de WIC (El Programa Especial De Nutrición Suplementaria Para Mujeres, Infantes Y Niños)
- ☐ CalWorks o bienestar
- ☐ Servicios de Black Infant Health
- ☐ Servicios de HPP-Homeless Prenatal Partnership
- ☐ Ninguna de las opciones anteriores

58) ¿Se considera usted una persona religiosa o espiritual?

- ◯ Mucho
- ◯ Moderadamente
- ◯ Ligeramente
- ◯ Para nada

59) Durante el embarazo, ¿qué tan satisfecho estaba usted con el apoyo dado por el padre de su bebé?

- ◯ Nada satisfecha
- ◯ Algo insatisfecha
- ◯ Algo satisfecha
- ◯ Muy satisfecha
- ◯ No aplica

Esta es la última sección de preguntas. Se enfoca en cuestiones demográficas.

Recuerde que usted puede saltar cualquier pregunta o parar de hacer la encuesta en cualquier momento. Usted también puede saltar preguntas si no aplican con su propia experiencia.

60) ¿Se considera Usted Hispano/a / Latinx?

- ◯ Yes
- ◯ No

61) ¿Cuáles categorías describen a Usted mejor? Marque todos que apliquen.

- ☐ Negro/a o Afroamericano/a
- ☐ Chino/a
- ☐ Filipino/a
- ☐ Otro/a Asiático/a
- ☐ Nativo/a Americano/a
- ☐ Nativo/a Hawaiano/a o otro/a Isleño/a Pacífico/a
- ☐ Blanco/a
- ☐ Otra categoría

62) Si escogió "otra categoría, por favor especifique:

____

63) ¿Cuáles países or regiones describen su origen racial/étnico mejor? Por favor escriba todos que apliquen.

Por ejemplo, Chino/a, Ruso/a, Chileno/a.

____

64) Durante su embarazo, ¿en qué barrio, código postal vivió? (Por favor, marque todos los barrios donde vivió)

- ☐ No vivo en ninguna de estos lugares
- ☐ Bayview, Hunters Point, 94124
- ☐ Chinatown, 94108
- ☐ Castro, Noe Valley, Corona Heights, 94114
- ☐ Excelsior, Ocean View, 94112
- ☐ Haight Ashbury, Hayes Valley, 94117
- ☐ Inner Richmond, Presidio, 94118
- ☐ Lake Merced, Lake Shore, 94132
- ☐ Marina, Cow Hollow, 94123
- ☐ Mission, Bernal Heights, 94110
- ☐ Nob Hill, Russian Hill, 94109
- ☐ North Beach, Telegraph Hill, 94133
- ☐ Parkside, Forest Hill, 94116
- ☐ Potrero Hill, Financial District, 94107
- ☐ Presidio, 94129
- ☐ Richmond, Sea Cliff, 94121
- ☐ Rincon Hill, 94105
- ☐ South of Market, 94103
- ☐ St Francis Wood, West Portal, 94127
- ☐ Sunset, 94122
- ☐ Tenderloin, Hayes Valley, 94102
- ☐ Twin Peaks, Diamond Heights, Glen Park, 94131
- ☐ Visitacion Valley, Portola, 94134
- ☐ Western Addition, Pacific Heights, 94115

65) ¿Qué tan seguro de la delincuencia cree que es su barrio?

- ○ Muy seguro
- ○ Algo seguro
- ○ Algo inseguro
- ○ Nada seguro
- ○ No lo sé

66) ¿En qué tipo de lugar vivió durante la mayor parte de su embarazo?

- ○ Apartamento
- ○ Remolque, casa móvil
- ○ Vivienda Pública, Sección 8
- ○ Con amigos (en el sofá o el piso)
- ○ Refugio, pensión
- ○ Coche, Van
- ○ En la calle o en una casa de campaña
- ○ Casa, adosado, condo
- ○ SRO, Motel o Hotel
- ○ Ninguna de las opciones anteriores

67) Sin contarse a usted, ¿cuántas personas viven con usted que tenían 18 años de edad o más?

- ○ 5+personas
- ○ 3-4 personas
- ○ 1-2 personas
- ○ 0 persona

68) Sin contarse a usted, ¿cuántas personas viven con usted que tenían 17 años de edad o menos?

- ○ 5+ personas
- ○ 3-4 personas
- ○ 1-2 personas
- ○ 0 personas

69) ¿El lugar donde vive usted tiene alguno de los siguientes? (Marque todas que apliquen.)

- ☐ Cocina completa
- ☐ Baño privado
- ☐ Ninguna de las opciones anteriores

70) Aquí hay algunas cosas que pueden pasarle a algunas personas durante sus embarazos. Por favor, díganos si alguna de estas cosas le sucedió durante este embarazo:

- ☐ Tuve que mudarme a una nueva dirección
- ☐ No tuve un lugar regular en donde podía dormir (tenía que andar de casa en casa)
- ☐ Estaba sin hogar (tuvimos que dormir fuera, o alojarme en un coche o un refugio)
- ☐ Ninguna de las opciones anteriores

71) ¿Cuánto tiempo ha vivido en los Estados Unidos?

- ◯ Menos de 5 años
- ◯ 5-10 años
- ◯ 10-15 años
- ◯ 15-20 años
- ◯ 20+ años
- ◯ Toda la vida

72) ¿Siente que tiene una comunidad cultural fuerte que le puede ayudar a encontrar servicios y recursos en el área de la Bahía?

- ◯ Sí, todo el tiempo
- ◯ Sí, algo del tiempo
- ◯ No, rara vez o nunca

73) ¿Cuál fue el total de sus ingresos familiares en 2016 antes de pagar impuestos?

Por favor, marque en el cuadro de abajo las sumas que incluyan todos los ingresos de su hogar, incluyendo sus ingresos, los ingresos de su esposo o pareja (si vivía juntos en 2016) y los de sus hijos.

Por favor, incluya todos sus ingresos, incluyendo el empleo, bienestar social (welfare), Seguro de Incapacidad (disability), Seguro de Desempleo, pagos para la manutención infantil (child support), intereses, dividendos y ayuda recibida de familiares.

- ◯ $0 to $16,000
- ◯ $16,001 to $20,000
- ◯ $20,001 to $24,000
- ◯ $24,001 to $28,000
- ◯ $28,001 to $31,000
- ◯ $31,001 to $36,000
- ◯ $36,001 to $39,000
- ◯ $39,001 to $47,000
- ◯ $47,001 to $55,000
- ◯ $55,001 to $59,000
- ◯ $59,001 to $63,000
- ◯ $63,001 to $71,000
- ◯ $71,001 to $78,000
- ◯ $78,001 to $83,000
- ◯ $83,001 to $94,000
- ◯ $94,001 to $107,000
- ◯ $107,001 to $110,000
- ◯ $110,001 to $126,000
- ◯ $126,001 to $142,000
- ◯ $142,001 or more

74) ¿Cuántas personas vivían de los ingresos de su hogar en 2016?

____

(Escriba un numero (ejemplo 3))

75) En general, durante este embarazo, ¿qué tan difícil fue para usted y su familia vivir con los ingresos que tenía?

- ◯ Muy dificil
- ◯ Algo dificil
- ◯ No muy dificil
- ◯ Para nada deficil

76) ¿Qué idioma usualmente habla en casa?

- ◯ Inglés
- ◯ Español
- ◯ Inglés y Español igualmente
- ◯ Cantonés
- ◯ Ruso
- ◯ Vietnamita
- ◯ Ucraniano
- ◯ Árabe
- ◯ Ninguna de las opciones anteriores

77) ¿Qué tan segura se siente llenando formularios médicos usted sola?

- ◯ Extremadamente
- ◯ Moderadamente
- ◯ Un poco
- ◯ Para nada

Ya ha terminado la encuesta.

¡Muchas gracias por tomar el tiempo para llenar esta encuesta! Usted ha contribuido positivamente a ayudar padres y niños del futuro.

Favor de revisar con la investigadora ahora, y explicara el sigiente paso.

78) Ya terminé con la encuesta.

- ◯ Sí

Hi _____, thank you for taking the time to participate in this study. We appreciate you taking part in the survey, the first part of the study. Now we are going to introduce the second part of the study, the interview. Before we begin, I’m going to give you a brief overview of this interview. We are talking to people who have experienced preterm labor or a preterm birth so that we can better understand your experiences leading up to your preterm labor. We know these can be difficult experiences to talk about, and we appreciate you taking the time out of your day for this interview. We are doing this because we want to improve how the public health system can better take care of pregnant parents in the future. We want to emphasize that your participation in this interview will not change the normal clinical care that you are receiving or plan to receive.

I will be asking you questions about your experiences leading up to coming to the hospital and about your experiences in the weeks leading up to coming to the hospital. We are also interested in learning about the advice you would give to other parents. The interview will take about 30 minutes to an hour.

There are no right or wrong answers to these questions. Please feel free to let me know at any time if there is a question you like, dislike, if you are confused, etc. It is also totally okay for you to not answer a question, or to stop this interview at any time.

If at any point you have questions, please do not hesitate to ask. Do you have any questions so far?

Ok, Let’s get started.

Study Aims:

- To understand the <u>chronic stressors and protective factors</u> associated with preterm birth risk in San Francisco
- To explore how <u>social determinants of health are associated with biological intermediates</u>, and how <u>prenatal care modifies the magnitudes of association</u>;

Notes for Memo:

The first few questions I'm going to ask are about what happened around the time when you first came in to be seen in labor and delivery. We are interested in learning more about what you felt and thought before coming into the hospital.<br><br>1. Think back to the day you came into the hospital on *(xx date).* Can you tell me about the day you came to the hospital with preterm labor….starting from the time you woke up.<br><br>1a. In the hours after you starting feeling *(yy symptom)*, what did you do? How did you know to do this?	PROBE:<br>• Work<br>• Abnormal/Normal feeling/ "I just knew"<br>• Provider's response to pt concerns/symptoms<br>• Braxton Hicks- what did they know about them?<br>• If patient mentions feeling anxious, ask about what made them anxious and how anxious they felt.<br><br>Example:<br>• Could you please tell me more about.....?<br>• What do you mean by...?<br>• What made you feel that way?<br>• Could you describe any particular emotions or worries you had?
Thank you for telling me that. Now I have a few specific questions to ask you about the days or days leading up to *(xx date)* when you came into the hospital.<br><br>2. You mentioned that you had felt *(yy symptom)* on the day you came into the hospital. **Did you have any other symptoms or sensations or feelings that day or the days and week before that?**<br>• Each time they names one, ask when it occurred in relationship to coming in to the hospital or clinic.<br>• Then ask: "Any other sensations or feelings that you can remember?"<br>If the participant says something like " I just felt something was wrong/Something didn't feel right?—ask them to elaborate<br><br>2a. During your pregnancy, **had you ever felt those symptoms or sensations before?**<br>• If yes:<br>o Did you think the symptoms around the time you came to the hospital were different than the ones experienced earlier in your pregnancy?<br>o What did you do when you felt them before?<br>• If they do not mention it: Did you go in to clinic or the hospital to get checked? What happened there?	PROBE:<br>• Abnormal/Normal feeling/ "I just knew"<br>• Provider's response to pt concerns/symptoms<br>• Braxton Hicks<br>• If patient mentions feeling anxious, ask about what made them anxious and how anxious they felt.<br><br>Example:<br>• Could you please tell me more about...?<br>• What do you mean by...?<br>• What made you feel that way?
3. Now I want to better understand **how you decided to come to the hospital or clinic** on *(xx date),* the day that you came in to be checked out because you were having *(yy symptoms).* For some people, it can be hard to figure out when they should go in to the hospital or clinic so we are interested in knowing more about how pregnant people make that decision. Can you think back to that day and tell me what made you decide to go to the hospital or clinic? What was your thought process around that decision?<br><br>3a. After starting to experience a new feeling of *(yy symptom)*, some patients come in to the hospital **soon after** the feeling/symptom starts, while others **wait** some time before coming in. What made you decide to come in when you did?	PROBE:<br>• Abnormal/Normal feeling/ "I just knew"<br>• Provider's response to pt concerns/symptoms<br>• Braxton Hicks<br>• Accessing services<br>• If patient mentions feeling anxious, ask about what made them anxious and how anxious they felt.

3b. Once you decided you were going to go in, were there any **challenges or delay**s in getting to the hospital?<br><br>3c. Did anything about your **prenatal care experience affect your decision** to come to the hospital?<br><br>3d. Was this the **first time you had ever gone in to labor and delivery** or "triage" to be seen? (ok if in other pregnancies).<br>i. If no, ask: "Why did you go in to be seen? When? What happened?<br>ii. And: "Did anything about that experience affect your decision to come to the hospital this time?	Example:<br>• Could you please tell me more about...?<br>• What do you mean by...?<br>• What made you feel that way?<br>• How did you feel about the medical teams decision?<br>• Could you describe any particular emotions or worries you had?
Thank you for sharing that part of your story with us. The second part of the interview focuses on the weeks leading up to your *(preterm labor / other reason)* and visit to the hospital. We are interested in better understand what pregnant parents are experiencing in the weeks leading up to the hospital. We will start with the day before you came to the hospital.<br><br>4. We'd like to ask you about what you remember, more generally, about the 24 hours before coming to the hospital. **Could you describe the 24 hours before you came to the hospital?** Feel free to go in as much detail as you want.<br>4a. Do you remember anything in particular about what you ate or drank?<br>4b. You mentioned *(revisit what they have described)*. Given what was happening in those 24 hours, how were you feeling?<br><br>5. Thank you for describing all of that. it was very helpful to hear it in your words. I'm also interested in knowing **what else was going on in your life** around the time you came in to the hospital on *(xx date*). If you think back to **the week** before you went to the hospital, what was happening that was stressful, hard for you to manage, or concerning within your family, work, relationship, home, or community life?<br><br>5a. If they start describing something and interviewer wants more details:<br>i. So this happened *(time when it happened)* OR When did this happen?...Could you tell me more about that experience?<br>ii. How did you cope with this stress? How difficult was it to cope with this stress?<br>iii. What was your support system like during this time? How did they support you?<br>iv. Were you comfortable talking to your prenatal care provider about these experiences? Can you tell me more about this?<br><br>5b. If they mention stress in one area (e.g. work), ask about the others.<br><br>5c. If they say nothing was happening:<br>v. Okay, so you felt like nothing stressful or hard to manage was happening week before you starting having *(preterm labor / other reason)*?<br>vi. How do you generally cope with stress?<br>vii. What was your support system like during this time? How did they support you?	Reminder: Clarify timeframe<br><br>PROBE:<br>• Accessing services (housing, medical including dental, employment, psych, etc)<br>• Work<br>• Braxton Hicks<br>• Housing<br>• If participant mentions mental health services or seeing therapist, probe a bit about how long they've had it/been in care/exacerbated by pregnancy, how they learned about the services, etc.<br>• If patient mentions feeling anxious, ask about what made them anxious and how anxious they felt.<br><br>Example:<br>• What was it like to access medical care before/during your pregnancy?<br>• Could you please tell me more about...?<br>• What do you mean by...?<br>• What made you feel that way?<br>• Could you describe any particular emotions or worries you had?

6. Thank you for sharing that with me. We just talked about what was happening in your life in the week before you came to the hospital. Now I am going to ask the same questions, but this time thinking back little further to the month before you came in. What was happening in your life then?<br><br>6a. Was anything different about your way of coping with stress then? About your support system?<br><br>6b. If needed, ask all the questions as in 5. above.	
The last part of the interview is focused on learning more about how parents can be better supported during their pregnancy. One of the goals of our study is to improve the education and care that pregnant parents are given both before and during their pregnancies. The following questions will help us learn how to best do that.<br><br>7. This question is about **medical care access**. What was it like to access medical care **before** your pregnancy? What was it like to access care **during** your pregnancy?<br><br>8. The next question is about different kinds of care and services:<br><br>8a. Did you have **high-risk prenatal care** (HROB)?<br>If yes: what did having HROB care mean to you?<br><br>8b. Did you have **group prenatal care** (Centering)?<br>If yes: What was it like?<br>If no: did anyone talk to you about group prenatal care? If yes: what made you decide not to participate?<br><br>8c. Did you have a **public health nurse**? If no: did anyone talk to you about public health nursing?<br>If yes: What was it like?<br>If no: did anyone talk to you about public health nursing? If yes: what made you decide not to participate?<br><br>If participant hasn't heard of these services: say "these are services that women are sometimes offered, and we want to learn more about how we can make these services more well-known and accessible for all patients."<br><br>9. Thank you for sharing those experiences and your thoughts. The next will help us get a better idea of the current state of prenatal care education. Before you *(gave birth / or came to the hospital with premature labor),* **what did you know** about preterm birth?<br><br>9a. **Where** did this knowledge come from?<br><br>9b. If the participant does not mention **prenatal care**, ask: Did you talk about **preterm labor/birth** in your prenatal care?<br><br>9c. Did you talk about **when to come to the hospital** in your prenatal care?<br><br>10. Now that you've had *(preterm birth / preterm labor / other experience),* **what advice or knowledge** would you give to other pregnant people?	PROBE:<br>• Accessing services<br>• If they say they previously had or were preterm themselves, did your provider know, include with medical hx?<br><br>Example:<br>• What was it like to access medical care before/during your pregnancy?<br>• Could you please tell me more about.....?<br>• What do you mean by...?<br>• What made you feel that way?

11. **What advice or knowledge** would you give to other health providers such as nurses, doctors, or midwives?<br><br>12. Are there **any other issues I haven't brought up** that you feel are important in understanding the experience and decision-making process around premature birth?	

Feedback (Keep recorder on):

1. Do you have suggestions for how to improve the interview?
2. Did you feel like the interview was: Too long, just right, too short, not sure/don't know
3. Was there anything you liked about the interview?
4. Is it important to study preterm birth: It's very important, somewhat important, it's not important
5. Do you have any other comments or suggestions?

Closing:

- Thank you so much for taking the time to share your story with me. Your experiences, thoughts, and opinions will contribute to helping improve the healthcare that other parents will receive.
- As a thank you for participating in our study, we wanted to give you this gift card.
- Thank you again. If you have any questions about the research study you can find the research team's contact information on the consent form you were given at the beginning of the study.

Hola _____, gracias por tomar el tiempo para participar en este estudio. Apreciamos que hayas tomado parte en la encuesta, que es la primera parte del estudio. Ahora vamos a introducir la segunda parte del estudio, la entrevista. Antes de empezar, le voy a dar una pequeña sinopsis de la entrevista. Estamos hablando con personas que han tenido experiencia con trabajo de parto pretérmino o que han dado a luz prematuramente para poder entender mejor sus experiencias antes del trabajo de parto pretérmino. Sabemos que es difícil hablar de estas experiencias, y apreciamos que se tome el tiempo para esta entrevista. Hacemos esto porque queremos mejorar la forma en la que el sistema de salud pública ofrezca cuidados a las personas embarazadas en el futuro. Queremos enfatizar que su participación en esta entrevista no afectará el cuidado médico normal que usted está recibiendo o planea recibir.

Le haré preguntas sobre sus experiencias antes de venir al hospital y sus experiencias en las semanas antes de venir al hospital. También estamos interesados en saber más sobre los consejos que ofrecería a otros padres. La entrevista durará aproximadamente 30 minutos hasta una hora.

No hay respuestas correctas o incorrectas a estas preguntas. Por favor avíseme en cualquier momento si hay alguna pregunta que le guste, que no le guste, si está confundida, etc. También está completamente bien si usted no responde a alguna pregunta, o si quiere parar la entrevista en cualquier momento.

Si tiene una pregunta en cualquier momento, por favor no dude en preguntar. ¿Tiene alguna pregunta hasta ahorita?

OK, vamos a comenzar.

Propósitos del Estudio:

- Entender los factores que causan estres crónico y los factores protectores asociados con el riesgo de nacimiento prematuro en San Francisco.
- Explorar como los determinantes de salud estan asociados con intermedios biológicos, y como el cuidado prenatal modifica las magnitudes de la asociación.

Notas para la Sección de Memorando:

<table>
<tr>
<td>Las primeras preguntas que le haré, son sobre lo que paso alrededor de cuando usted primero fue atendida en el area de maternidad. Nos interesa saber más sobre lo que sintió y pensó antes de venir al hospital.

1. Piense en el día que llegó al hospital en (xx fecha). Puede contarme que pasó el día que vino al hospital para el trabajo de parto… empezando con el momento en que despertó.

1a. En las horas después de empezar de sentir (yy síntoma), ¿qué hizo usted? ¿Cómo usted sabía hacer esto?</td>
<td>EXPLORA:
• Trabajo
• Sentimientos abnormales/normales, “Yo justo supe”
• Respuestas de proveedores médicos a los preocupaciones/síntomas del paciente
• Contracciones falsas (“Braxton Hicks”)—que sabía sobre este tema?
• Si el/la paciente menciona que se sentía preocupado/a, pregunta que le hizo sentir eso y que nivel de preocupación/ansiedad se sentía.

Ejemplos:
• Por favor, ¿me puede decir más sobre…?
• ¿Qué quería decir cuando dijo…?
• ¿Qué le hacía sentirse en esta manera?
• ¿Me puede describir algunas emociones o preocupaciones que tenía?</td>
</tr>
<tr>
<td>Gracias por contarme eso. Ahora tengo algunas preguntas específicas que hacerle sobre los días anteriores al (xx fecha) cuando vino al hospital.

2. Usted mencionó que sentía (yy síntoma) el día que vino al hospital. ¿Tuvo alguno otro síntoma o sensación ese día, o los días y la semana anteriores?
• Cada vez que nombre uno, pregunte cuando ocurrió en relación al viaje a la clínica u hospital.
• Luego pregunta: “¿Alguna otra sensación o sentimiento que pueda recordar?”
• Si la participante dice algo como “Sentí que algo no estaba bien/Algo no se sentía bien,” pida que le den más detalles.

2a. Durante su embarazo, ¿había sentido alguna vez estos síntomas o sensaciones antes?
• Si la respuesta es sí:
o ¿Pensó que los síntomas alrededor de cuando vino al hospital eran diferentes a los que experimentó anteriormente durante el embarazo?
o ¿Que había hecho cuando los sintió anteriormente?
• Si no lo mencionan: ¿Acudió a la clínica u hospital para un chequeo? ¿Qué pasó allí?</td>
<td>EXPLORA:
• Sentimientos abnormales/normales, “Yo justo supe”
• Respuestas de proveedores médicos a los preocupaciones/síntomas del paciente
• Contracciones falsas (“Braxton Hicks”)—que sabía sobre este tema?
• Si el/la paciente menciona que se sentía preocupado/a, pregunta que le hizo sentir eso y que nivel de preocupación/ansiedad se sentía.

Ejemplos:
• Por favor, ¿me puede decir más sobre…?
• ¿Qué quería decir cuando dijo…?
• ¿Qué le hacía sentirse en esta manera?</td>
</tr>
<tr>
<td>3. Ahora quiero entender mejor como decidió venir al hospital o clínica en (xx fecha), el día que vino para un chequeo porque usted sentía (yy síntomas). Para algunas personas, puede ser difícil saber cuando deberían ir al hospital o clínica, entonces estamos interesados en saber más sobre como toman esas decisiones las personas embarazadas. ¿Puede recordar ese día y decirme que le hizo decidir acudir al hospital o clínica? ¿Cuál fue su proceso de pensamiento en torno a esa decisión?

3a. Después de sentir una sensación nueva de (yy síntomas), algunos pacientes deciden ir al hospital pronto después del comienzo del síntoma, mientas otros esperan un rato antes de ir. ¿Qué le hizo decidir a ir cuando usted fue?</td>
<td>EXPLORA:
• Sentimientos abnormales/normales, “Yo justo supe”
• Respuestas de proveedores médicos a los preocupaciones/síntomas del paciente
• Contracciones falsas (“Braxton Hicks”)—que sabía sobre este tema?
• Accediendo servicios
• Si el/la paciente menciona que se sentía preocupado/a, pregunta que le hizo sentir eso y que nivel de preocupación/ansiedad se sentía.</td>
</tr>
</table>

3b. Una vez que decidió acudir, ¿hubo **dificultades** o **retrasos** para poder llegar al hospital?<br><br>3c. ¿Hubo algún aspecto de sus **experiencias con el cuidado prenatal que afectara su decisión** de acudir al hospital?<br><br>3d. ¿Fue esta **la primera vez que había acudido a maternidad** o "triaje" para ser atendida? (OK si sucedió en otros embarazos).<br>i. Si no, preguntar: "¿Porque acudió a que la vieran? ¿Cuando? ¿Que pasó?<br>ii. Y: "¿Alguna cosa sobre esa experiencia afectó su decision de venir al hospital esta vez?"	Ejemplos:<br>• Por favor, ¿me puede decir más sobre…?<br>• ¿Qué quería decir cuando dijo…?<br>• ¿Qué le hacía sentirse en esta manera?<br>• ¿Cómo se sentía sobre la decisión del equipo médico?<br>• ¿Me puede describir algunas emociones o preocupaciones que tenía?
Gracias por compartir esa parte de su historia con nosotros. La segunda parte de la entrevista se enfoca en **las semanas anteriores** a su (*trabajo de parto pretérmino / otra razón)* y su visita al hospital. Nos interesa entender mejor lo que los padres que están esperando un bebé experimentan en las semanas anteriores a la visita al hospital. Empezaremos con **el día** antes de que usted llegara al hospital.<br><br>4. Quisiéramos preguntarle que recuerda, generalmente, sobre las 24 horas antes de venir al hospital. **¿Podría describir las 24 horas antes de que viniera al hospital?** Puede elaborar con tantos detalles como usted quiera.<br><br>4a. ¿Recuerda algo en particular sobre lo que comió o bebió?<br><br>4b. Usted mencionó (*revisar lo que hayan descrito*). Dado todo lo que estaba pasando en esas 24 horas, ¿cómo se sentía?<br><br>5. Gracias por describir todo eso. Ayuda mucho escucharla en sus propias palabras. También tenemos interés en saber **que otras cosas estaban ocurriendo en su vida** alrededor de cuando vino al hospital en (*xx fecha*). Si recuerda **la semana antes** de acudir al hospital, ¿que estaba pasando que fuera **estresante**, difícil para usted, o en cuanto a su familia, trabajo, relación, casa o vida comunitaria?<br><br>5a. Si empieza a describir algo y quiere más detalles:<br>i. Entonces esto sucedió (*hora en que sucedió*) O ¿Cuando sucedió esto?… ¿Me podría decir más sobre esa experiencia?<br>ii. ¿Cómo usted aguantaba (o sobrellevaba) este estrés? ¿Qué difícil fue de aguantar este estrés?<br>iii. ¿Cómo fue su sistema de apoyo durante ese tiempo?<br>iv. ¿Usted se sentía cómoda hablando con su proveedor de cuidado prenatal sobre estas experiencias? ¿Me podría decir más sobre esto?<br><br>5b. Si menciona estrés en una area (por ejemplo, trabajo), pregunta sobre las otras.<br><br>5c. Si dice que no estaba pasando nada:<br>v. Ok, ¿entonces usted sintió que no pasaba nada estresante o difícil para usted en la semana antes de que comenzara a tener (*trabajo de parto / otra razón por venir)*?<br>vi. ¿Cómo usted aguantaba (o sobrellevaba) estrés generalmente?	RECUERDO: Clarificar la cronología<br><br>EXPLORA:<br>• Accediendo servicios (para casas, medicos incluyendo dentales, empleos, psiquiatría, etc)<br>• Trabajo<br>• Contracciones falsas ("Braxton Hicks")—que sabía sobre este tema?<br>• Hogar<br>• Si la participante mencionó servicios de salud mental o visitando a un terapeuta, explora: por cuanto tiempo ha tenido este asunto, contacto con cuidado, empeorado por embarazo, como aprendió sobre servicios…<br>• Si el/la paciente menciona que se sentía preocupado/a, pregunta que le hizo sentir eso y que nivel de preocupación/ansiedad se sentía.<br><br>Ejemplos:<br>• ¿Cómo fue la experiencia de acceder servicios médicos antes de/durante su embarazo?<br>• Por favor, ¿me puede decir más sobre…?<br>• ¿Qué quería decir cuando dijo…?<br>• ¿Qué le hacía sentirse en esta manera?<br>• ¿Me puede describir algunas emociones o preocupaciones que tenía?

vii. ¿Cómo fue su sistema de apoyo durante ese tiempo?<br><br>6. Gracias por compartir eso conmigo. Acabamos de hablar sobre lo que estaba pasando en su vida durante la semana antes de su visita al hospital. Ahora voy a preguntarle las mismas preguntas, pero esta vez pensando en **el mes anterior** a su visita al hospital. ¿Qué estaba pasando en su vida durante este tiempo?<br><br>6a. ¿Había algo que fue diferente sobre su manera de aguantar estrés durante este tiempo? ¿Había algo que fue diferente sobre su sistema de apoyo?<br><br>6b. Si es necesario, pregunta las preguntas en 5.	
La última parte de la entrevista se enfoca en aprendiendo más sobre como podemos **dar mejor apoyo** a los padres durante su embarazo. Uno de los objetivos de nuestro estudio es mejorar la educación y el cuidado que reciben los padres que están esperando un bebé. Las siguientes preguntas nos ayudarán a aprender la mejor forma de hacer esto.<br><br>7. Esta pregunta es sobre acceso a servicios médicos. ¿Cómo fue la experiencia de acceder servicios médicos **antes de** su embarazo? ¿Cómo fue la experiencia de acceder servicios médicos **durante** su embarazo?<br><br>8. La siguiente pregunta es sobre diferentes tipos de cuidado y servicios:<br>8a. ¿Recibió usted cuidado prenatal de **alto riesgo**?<br>Si la respuesta es **sí:** ¿Qué lo significa este cuidado para usted?<br><br>8b. ¿Recibió usted cuidado prenatal de **en grupo** (Centering)?<br>**Sí:** ¿Cómo fue esa experiencia?<br>**No:** ¿Alguien habló con usted sobre cuidado en grupo? Si sí, ¿qué le hizo decidir no participar?<br><br>8c. ¿Recibió usted servicios de un **enfermero de salud** p**ública**?<br>**Sí:** ¿Cómo fue esa experiencia?<br>**No:** ¿Alguien habló con usted sobre la enfermería de salud pública? Si sí, ¿qué le hizo decidir no participar?<br><br>Si el participante no sabe de estos servicios, diga: "Estos son servicios que a veces se ofrece a mujeres, y queremos aprender más sobre como podemos hacer estos servicios más conocidos y accesibles para todos los pacientes."<br><br>9. Gracias por compartir esas experiencias y sus ideas. La siguiente pregunta nos ayudará a tener una mejor idea del estado actual de la educación de cuidado prenatal. Antes de (*dar a luz / venir al hospital con xx*), **¿qué sabía sobre el nacimiento prematuro?**<br><br>9a. ¿Dónde aprendió esta información?<br><br>9b. Si el participante no menciona el cuidado prenatal, diga: ¿Habló sobre el nacimiento o el trabajo de parto prematuro en su cuidado prenatal?<br><br>9b. ¿Habló sobre cuando debe ir al hospital en su cuidado prenatal?	EXPLORA:<br>• Accediendo servicios<br>• Cuidado prenatal de alto riesgo—¿la participante lo recibió? ¿ella pensa que lo recibió? ¿qué lo significa para usted?<br>• Si dice que tenía un parto pretérmino en el pasado o que estaba premature ella misma, pregunta: ¿su proveedor médico lo sabía?<br><br>Ejemplos:<br>• ¿Cómo fue la experiencia de acceder servicios médicos antes de/durante su embarazo?<br>• Por favor, ¿me puede decir más sobre…?<br>• ¿Qué quería decir cuando dijo…?<br>• ¿Qué le hacía sentirse en esta manera?

10. Ahora que ya ha tenido (*parto prematuro / trabajo de parto pretérmino / otra experiencia*), ¿**cuales consejos o conocimientos** le daría a otras personas embarazadas?<br><br>11. ¿**Cuales consejos o conocimientos** le daría a proveedores médicos como enfermeras, doctores, o parteras?<br><br>12. ¿Hay **algún otro asunto que no he mencionado** que usted siente que es importante que nosotros entendamos sobre la experiencia y la toma de decisiones relacionadas al parto prematuro?	

Ideas / Reacciones a la entrevista: (Dejar prendida la grabadora):

1. ¿Tiene alguna sugerencia sobre como mejorar la entrevista?
2. ¿Usted siente que la entrevista fue: demasiado larga, justo lo correcto, demasiado corta, no sabe o no está segura?
3. ¿Hay algo que le haya gustado sobre la entrevista?
4. ¿Qué piensa: Es importante estudiar el nacimiento prematuro: es muy importante, algo importante, no es importante?
5. ¿Tiene algún otro comentario o sugerencia?

En conclusión:

- Muchísimas gracias por tomar el tiempo para compartir su historia conmigo. Sus experiencias, pensamientos, y opiniones contribuirán a ayudarnos a mejorar el servicio médico que recibirán otros pacientes.
- Para darle las gracias por su participación en nuestro estudio, queremos ofrecerle esta tarjeta de regalo.
- Gracias nuevamente. Si tiene alguna pregunta sobre este estudio de investigación puede encontrar información sobre como comunicarse con el equipo de la investigación en el formulario de consentimiento que se le dio al principio del estudio.
